# Investigating Motor Coordination Using BXD Recombinant Inbred Mice to Model the Genetic Underpinnings of Developmental Coordination Disorder

**DOI:** 10.1111/gbb.70014

**Published:** 2025-03-12

**Authors:** Jeffy Rajan Soundara Rajan, Kamaldeep Gill, Eric Chow, David G. Ashbrook, Robert W. Williams, Jill G. Zwicker, Daniel Goldowitz

**Affiliations:** ^1^ Department of Medical Genetics University of British Columbia Vancouver Canada; ^2^ Centre for Molecular Medicine and Therapeutics University of British Columbia Vancouver Canada; ^3^ British Columbia Children's Hospital Research Institute Vancouver Canada; ^4^ Rehabilitation Sciences University of British Columbia Vancouver Canada; ^5^ Department of Genetics, Genomics and Informatics University of Tennessee Health Science Center Memphis Tennessee USA; ^6^ Department of Occupational Science & Occupational Therapy University of British Columbia Vancouver Canada; ^7^ Department of Pediatrics University of British Columbia Vancouver Canada

**Keywords:** BXD recombinant inbred strains, candidate genes, developmental coordination disorder, gait analysis, heritability, motor coordination, neonatal reflexes, open field, quantitative trait analysis, rotarod

## Abstract

The fundamental skills for motor coordination and motor control emerge through development. Neurodevelopmental disorders such as developmental coordination disorder (DCD) lead to impaired acquisition of motor skills. This study investigated motor behaviors that reflect the core symptoms of human DCD through the use of BXD recombinant inbred strains of mice that are known to have divergent phenotypes in many behavioral traits, including motor activity. We sought to correlate behavior in basic motor control tasks with the known genotypes of these reference populations of mice using quantitative trait locus (QTL) mapping. We used 12 BXD strains with an average of 16 mice per group to assess the onset of reflexes during the early neonatal stage of life and differences in motor coordination using the tests for open field, rotarod, and gait behaviors during the adolescent/young adulthood period. Results indicated significant variability between strains in when neonatal reflexes appeared and significant strain differences for all measures of motor coordination. Five strains (BXD15, BXD27, BXD28, BXD75, BXD86) struggled with sensorimotor coordination as seen in gait analysis, rotarod, and open field, similar to human presentation of DCD. We identified three significant quantitative trait loci for gait on proximal Chr 3, Chr 4, and distal Chr 6. Based on expression, function, and polymorphism within the mapped QTL intervals, seven candidate genes (*Gpr63, Spata5, Trpc3, Cntn6, Chl1, Grm7, Ogg1*) emerged. This study offers new insights into mouse motor behavior, which promises to be a first murine model to explore the genetics and neural correlates of DCD.

## Graphical abstract

Core symptoms of human DCD modeled in BXD recombinant inbred strains of mice. Significant strain differences for all measures of motor coordination were found. Three significant quantitative trait loci and seven candidate genes for gait were identified. Study offers new insights into mouse motor behavior and the first murine model to explore the genetics and neural correlates of DCD.

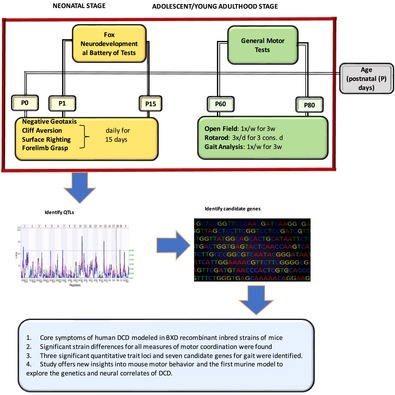

## 1 Introduction

The fundamental skills for motor coordination and motor control largely emerge through development, from infancy to late childhood to adulthood [1]. When this developmental progression is perturbed as a result of genetic mutations and/or altered brain development, neurodevelopmental disorders can emerge [1]. Neurodevelopmental disorders can cause atypical cognitive functioning, intellectual impairment, and/or motor developmental delays [1].

Developmental coordination disorder (DCD) is a neurodevelopmental disorder affecting 5%–6% of school‐aged children [2]. DCD is characterized by deficits in postural control, sensorimotor coordination, and motor learning that are not due to a neurological disorder, such as cerebral palsy [2]. The motor deficits associated with DCD significantly affect a child's ability to carry out activities of daily living, such as tying shoelaces, getting dressed, printing, or riding a bicycle [3, 4]. Motor performance of children with DCD is more variable, less accurate, and slower than typically developing children [3]. While DCD is reported to be more prevalent in boys in clinical samples (3:1–7:1) [5], population‐based studies indicate a more equivalent sex distribution (1:1.9) [6]. Children with DCD typically do not outgrow these motor impairments, rather they persist into adolescence and adulthood [7].

DCD is a multifactorial disorder with unclear etiology, partly due to its heterogeneous nature. Research to date has suggested that genetics, family history, and altered brain development play a role in the phenotypic variability seen in DCD [8, 9]. Mouse models are a commonly used experimental platform [10] to explore such heterogeneous human disorders in order to study the genetic, molecular, and behavioral aspects of human diseases. In modeling a complex disorder like DCD in the mouse, it is necessary to examine DCD‐relevant behaviors across a spectrum of variation. The use of reference panels of mice, such as recombinant inbred (RI) strains, has proven to be a powerful approach to our understanding of a broad spectrum of disorders [11]. The BXD family, currently the largest and best characterized mammalian reference population, is composed of 140 strains [12]. Additionally, the behavioral repertoire of BXD RI mice is diverse and reflects the substantial differences between the progenitor lines of the BXD strains, C57BL/6J and DBA/2J mice [13].

**FIGURE 1:**
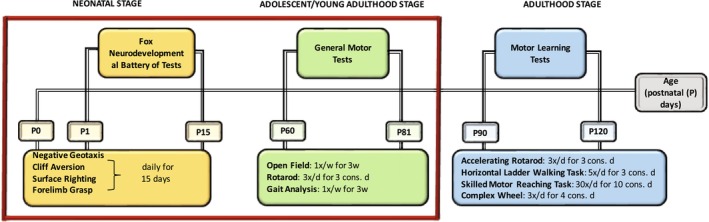
Workflow of the behavioral testing in Postnatal Day (P) P1‐120. All three phases of testing are proposed based on the DCD‐like behavior [d, days; w, week; cons, consecutive]. On the left‐hand side, outlined by the red box, are the tests used in this paper.

To capture the motor variability seen in BXD RI mice, we devised a battery of motor behavior tasks (Figure 1) to mimic the motor deficits observed in human DCD (see Gill et al. [10] for details). Using this approach, we can begin to identify candidate regions of chromosomes that harbor genes that underlie the behaviors of interest in DCD using quantitative trait loci (QTL) analysis. In this paper, we focus on the variation of motor coordination phenotypes and corresponding QTLs in BXD strains of mice and assess whether we have access to variations in phenotypes that are relevant to DCD and correlations with genotypes.

## 2 Materials and Methods

### 2.1 Animals

The BXD breeding colony used to generate mice for this study was established from mice purchased from the Jackson Laboratory (Bar Harbor, ME) and housed at the Transgenic Animal Facility, Center for Molecular Medicine and Therapeutics, University of British Columbia. Table 1 presents a summary of the total number of mice, sample size per strain, number of litters, and number of males and females in each strain. The strains included in the study were C57BL/6J (B6), DBA/2J (DBA), and 12 BXD strains (BXD1, BXD15, BXD27, BXD28, BXD32, BXD40, BXD45, BXD65a, BXD69, BXD75, BXD81, BXD86) for each of the behavioral measures. All animals were housed in same sex groups of 2–5 at a temperature of 22.5°C, with a 12:12 h light–dark cycle (lights on at 6:00 am). Food and water were freely available, and bi‐weekly cage changes took place. All testing took place during the morning hours (light phase) between 8:00 and 12:00 by co‐authors K.G. and J.S.R. All experiments were conducted in accordance with the guidelines of the Canadian Council of Animal Care, and all protocols were approved by the University of British Columbia Animal Care Committee (ACC).

**TABLE 1:** Number of mice tested for each motor co‐ordination task.

		Total number of mice								
ID	Strain	# of Litters	Fox battery		Rotarod		Open field		Gait analysis	
1	C57BL/6J	3	18	M: 9	18	M: 9	18	M: 9	18	M: 9
				F: 9		F: 9		F: 9		F: 9
2	DBA/2J	4	18	M: 9	18	M:9	18	M:9	18	M:9
				F: 9		F: 9		F: 9		F: 9
3	BXD1	4	18	M:9	18	M: 9	18	M: 9	18	M: 9
				F: 9		F: 9		F: 9		F: 9
4	BXD15	3	18	M: 9	18	M: 9	18	M: 9	18	M: 9
				F: 9		F: 9		F: 9		F: 9
5	BXD27	3	18	M: 9	18	M: 9	18	M:9	18	M:9
				F: 9		F: 9		F: 9		F: 9
6	BXD28	3	18	M: 9	18	M: 9	18	M: 9	18	M: 9
				F: 9		F: 9		F: 9		F: 9
7	BXD32	4	24	M: 12	24	M: 12	24	M: 12	24	M: 12
				F: 12		F: 12		F: 12		F: 12
8	BXD40	4	24	M: 12	24	M: 12	24	M: 12	24	M: 12
				F: 12		F: 12		F: 12		F: 12
9	BXD45	3	16	M: 8	16	M: 8	16	M: 8	16	M: 8
				F: 8		F: 8		F: 8		F: 8
10	BXD65a	4	27	M: 12	27	M: 12	27	M: 12	27	M: 12
				F: 15		F: 15		F: 15		F: 15
11	BXD69	4	25	M: 12	25	M: 12	25	M: 12	25	M: 12
				F: 13		F: 13		F: 13		F: 13
12	BXD75	3	22	M: 11	22	M: 11	22	M: 11	22	M: 11
				F: 11		F: 11		F: 11		F: 11
13	BXD81	5	18	M: 9	18	M: 9	18	M: 9	18	M: 9
				F: 9		F: 9		F: 9		F: 9
14	BXD86	3	23	M: 13	23	M: 13	23	M: 13	23	M: 13
				F: 10		F: 10		F: 10		F: 10

Abbreviations: F, denotes female; M, denotes male.

The selection of each BXD strain was based on documented behavioral phenotypes and structural brain differences in the BXD population. Common assays, such as rotarod, open field, dowel test, and MRI findings were used to select strains that demonstrated core phenotypes that sat at the extremes and the mid‐point for balance (latency to fall on rotarod, dowel test), motor coordination (spontaneous locomotion in open field), and cerebellar volume as measured by MRI. Thus, strains were selected based on behavioral performance on motor coordination and learning tasks that ranged from poor to very good, in relation to human DCD phenotypes (Table 2) [13]. Further, BXD69, BXD75, BXD81, and BXD86 were selected for their performance variability ranging from poor to very good on the rotarod [14]. Other strains, BXD32, BXD45, and BXD65a, were selected either due to observed variation in balance abilities on the dowel test and/or spontaneous locomotion in the open field [13, 14]. BXD 1, BXD15, BXD27, BXD28, and BXD40 were selected due to their variation in cerebellar volume [10, 15], given the hypothesized role of the cerebellum in human DCD [15].

**TABLE 2:** Selection of BXD strains based on motor‐related traits.

GeneNetwork ID	Phenotype	Strain(s)
10,921	Cerebellar volume	BxD1/TyJ
		BxD15/TyJ
		BxD27/TyJ
10,921; 11,819	Cerebellar volume; dowel test	BxD28/TyJ
11,819	Dowel test	BxD32/TyJ
10,921	Cerebellar volume	BxD40/TyJ
11,014	Open field behavior	BxD45/RwwJ
		BxD65a/RwwJ
11,004	Rotarod performance	BxD69/RwwJ
		BxD75/RwwJ
11,014; 11,005	Open field behavior; improvement in rotarod	BxD81/RwwJ
11,004; 11,819	Rotarod performance; dowel test	BxD86/RwwJ

#### 2.1.1 Fox Neurodevelopmental Battery (P1‐15)

The Fox Neurodevelopmental Battery [16] was used to ensure mouse pups were developing typically with respect to nervous system maturation. Beginning at postnatal day 1 (P1), mouse pups were examined daily for emergence of developmental milestones. Testing was performed at the same time each day. Mouse tails were marked by indelible ink at P1 to allow us to identify each mouse over the 15‐day testing period.

Prior to daily testing, cages with the mother and pups were moved to the testing room, and mice were habituated to the room for approximately 30–60 min before testing. After habituation, the pups were removed from the home cage and placed on small amounts of nesting material, which was placed on a heating pad set at 37°C. Each day the pups were weighed, examined for eye opening, ear unfolding, and fur appearance, and then tested for surface righting, negative geotaxis, cliff aversion, and forelimb grasp. The order of testing was randomized each day for each litter to limit test order interaction.

##### 2.1.1.1 Righting Reflex

The pups were held gently in the supine position and then released while recording the time it took the pups to right themselves onto their abdomens with all four paws touching the measurement surface [16]. If pups did not respond within 30 s, the trial was terminated; each pup received up to three trials to right itself. This reflex is equivalent to the labyrinthine and body righting reflexes in infants [17].

##### 2.1.1.2 Negative Geotaxis

The mouse pups were placed on a 45° slope with their heads pointing down the incline; the time it took the pup to turn 180° to the “head up” position was measured [16]. If the pup lost its footing and slipped down the incline or was unable to complete the task within 30 s, the trial was terminated and a second trial was given, for up to three trials each day. A successful trial terminated the day's testing. Negative geotaxis can be related to the body righting reflexes, such as body‐on‐body reflex and segmental rolling reflex in human infants [17].

##### 2.1.1.3 Cliff Aversion

The mouse pups were placed on the edge of a cliff with the forepaws and snout over the edge; the time taken for the pup to turn and begin to crawl away from the edge was recorded [16]. If the pup slipped off the surface or if the test was not completed within 30 s, the pup was provided with another trial, for up to three trials each day. A successful trial terminated the day's testing. Although not directly related to human infant reflexes, cliff drop aversion can be related to the “parachute” protective extension reflex in humans [17].

##### 2.1.1.4 Forelimb Grasp

The pups were held about 5 cm above the testing surface; the forelimbs were stroked with a blunt instrument, which triggered the pup to flex the forepaws to grasp the instrument [16]. The length of time the pup gripped the rod was recorded. If the pup fell off immediately or could not grip the rod for a minimum of 1 s, the test was repeated for up to three trials each day. A successful trial terminated the day's testing. This reflex can be directly translated to the palmer grasp reflex in infants.

#### 2.1.2 General Motor Testing (P81‐90)

The order of testing for all general motor tasks was randomized for each litter on each testing session to limit the test order interaction and the effect of fatigue on a given performance.

##### 2.1.2.1 Rotarod

General motor coordination and balance was assessed by the rotarod at P60. All mice were trained on the Ugo Basil Rotarod with a rod diameter of 3 cm and a constant speed of 18 rpm over 2 min. Mice were tested three times a day with 30 min inter‐trial duration for three consecutive days for each mouse. Latency was measured as the time from the beginning of the trial until the time that the mouse fell off the rod and onto the lever, which stopped the timer. Mouse holding on to the rod and rotating for three consecutive rotations were scored as a fall. If a mouse fell off within 5 s from the beginning of the trial, the mouse was given a retrial and the previous one was not counted. The maximum trial length was 120 s.

##### 2.1.2.2 Open Field

General locomotor activity was assessed by open field activity at P60. Mice were placed in the center of an open field chamber constructed from Plexiglas with the dimensions of 50 cm × 50 cm × 12 cm. The center of the area was defined as a 20 cm × 20 cm central square. The activity of each mouse was digitally recorded for 10 min and subsequently analyzed by Noldus Ethovision XT Software (Noldus Information Technology). Noldus Ethovision XT is a video tracking software that can detect an animal in a video file or from a live video feed, distinguish it from its background, and track its movements and behavior. For this model, time spent in the center of the open field as opposed to the periphery of the open field, distance traveled, time spent moving, time spent not moving, and the velocity were measured.

##### 2.1.2.3 Gait Analysis

Gait was assessed using a digital gait analysis software as published by Mendes et al. [18] A transparent walkway apparatus with an angled mirror was constructed as per the design described by Mendes et al. [18] Videos were captured using a CM3‐U3‐13Y3C 1/2 “Chameleon3 color camera with Fujinon Dv3.4x3.8sa‐Sa1 1/2” 3.8‐13 mm F1.4 Dc Auto‐Iris Vari‐Focal C‐Mount lens. This method was based on the reflection of light within a transparent material through an optical effect termed total internal reflection. Foot contact disrupts this effect causing “frustrated total internal reflection” (fTIR), a technique that generates scattered light that is detected by a high‐speed video camera. The floor is made of acrylic plastic surrounded by LED lights, thus producing a touch sensor which is viewed using a mirror placed at 45° angle below the walking surface.

Gait analysis was performed on a weekly basis for a total of 3 weeks to measure comprehensive movement features, including static, stance, and dynamic parameters. The main advantage of this method is that differences in mouse gait parameters can be related to problems seen in children's gait over time. On the testing day, mice were placed on the walking apparatus and allowed to traverse freely across a narrow walkway for a minimum of 2 s. Recorded videos were analyzed using MATLAB and MouseWalker (an integrated hardware and software system that provides a comprehensive and quantitative description of walking behavior of rodents). In this study, the following phenotypic measures were used to address movement differences in balance and coordination: (1) body speed (mm/s): walking speed; (2) stance duration (s): time of initial contact with the surface at touchdown by the rostral paw to toe off prior to lift off; (3) swing duration (s): time the limb(s) spend not in contact with the surface, just prior to touchdown; (4) step cycle (s): duration of heel strike of one paw to the heel strike of the same paw in preparation for the next step; (5) duty factor: fraction of step cycle where the limb is in the stance phase (stance duration/period); (6) leg combination index (%): percentage of total step cycle spent in no swing, single‐leg swing, diagonal‐leg swing, lateral‐leg swing, front or hind swing, three‐leg swing, or all‐legs swing; (7) posterior extreme position (body units): position of the paw relative to the center of the body from paw touchdown to the end of the stance phase.

### 2.2 Behavioral Data Analysis

Statistical analysis was carried out using IBM SPSS Statistics Version 25.0. Data were analyzed separately for each test by one‐way ANCOVA using strain as an independent variable and body weight and sex as covariates. Post hoc comparisons were performed when appropriate using the Bonferroni method. To control for potential litter effects, we used the strain averages for each of the respective variables. Further, using strain average increases the power to detect the genetic effects [19].

An average of all trials per day for each task was taken for each of the testing days. The effect of the strain over the several testing days was explored using a two‐way ANCOVA with body weight and sex as covariates. To examine motor performance improvements, a difference score was calculated (mean of Day 3 or 4—mean of Day 1). All measures are reported as the mean ± S.E.M. and the level of significance reported for all comparisons is *p* < 0.05. For the Fox Neurodevelopmental Battery, days were treated as a dependent, random effect variable in our model. Day was treated as a variable in the repeated measures within subject ANCOVA.

Heritability is the proportion of phenotypic variance that is explained by genetic differences. In fully homozygous strains, such as the BXD, additive genetic variance (*V*_*a*_) is the main source of differences between strains, and our estimates can be considered close to what is typically called narrow‐sense heritability (*h* ^2^). *h* ^2^ was estimated as the fraction of variance explained by strains in a simple ANOVA model. We calculated locus effect size as the proportion of heritable variation that was explained by the peak marker at that QTL region, again using an ANOVA model. We have greater power to detect QTL when heritability is high (i.e., more of the total phenotypic variance is explained by genometype) and when locus effect size is high (i.e., when more of the heritable variation is explained by a single locus).

### 2.3 QTL Mapping

The behavioral trait data were uploaded into GeneNetwork (www.genenetwork.org), an open‐access database which contains genotypes for the BXD family and phenotypic information. For each phenotype, we mapped QTLs using the fast linear regression equations of Haley and Knott [20].

The likelihood ratio statistic (LRS) was computed to assess the strength of genotype–phenotype associations. When appropriate, outlier values for specific traits were winsorized. A test of 2000 permutations was performed to evaluate the statistical significance of associations and identify suggestive (*p* < 0.63) and significant (*p* < 0.05) QTLs. The bootstrap test was implemented to estimate the confidence interval by generating multiple bootstrap datasets by randomly selecting strains with substitution from the original RI set. Confidence intervals around the significant LRS score peaks were calculated using 1.5 logarithm of the odds (LOD) confidence interval.

### 2.4 Candidate Gene Analysis

Using the online tools in Mouse Genome Informatics (MGI), PubMed, and GeneNetwork, the genes within the QTL confidence interval were evaluated based on three criteria: (1) gene expression within brain and skeletal muscle region of interests; (2) relevant gene functions from previous literature; and (3) the presence of polymorphisms (i.e., non‐synonymous SNPs), as the phenotypic difference is assumed to be associated by genetic variation and considered as good candidate genes. The expression, functional, and phenotypic information for each of the genes located within the significant QTLs [21, 22] was assessed using MGI database, and PubMed. Second, literature searches were conducted to determine if each gene under the significant QTL peak had a previously reported role in motor skills and learning. Lastly, GeneNetwork variant browser was used to identify SNP variants specifically nonsynonymous polymorphism between parental haplotypes. We acknowledge that these are imperfect criteria but they serve as starting points for the winnowing of candidate genes.

## 3 Results

The phenotypic analysis of BXD mice proceeded as depicted in Figure 1.

### 3.1 Neurodevelopmental Battery

**FIGURE 2:**
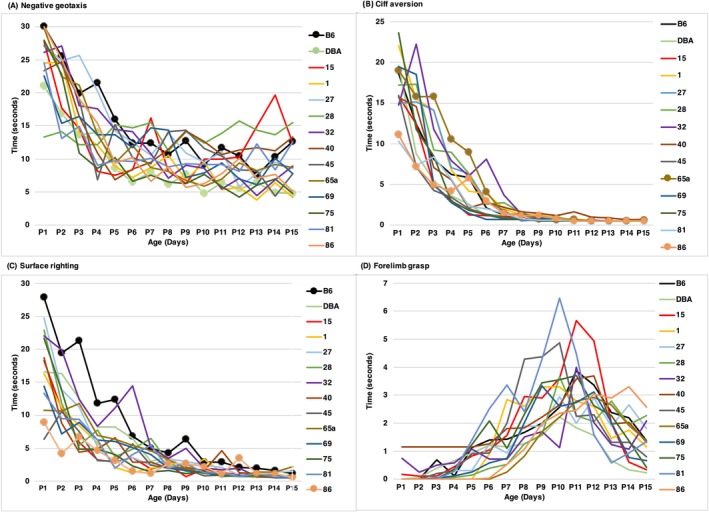
Graphs illustrate strain response time on Fox Neurodevelopmental Battery over the first 15 postnatal days. (A) Negative geotaxis; (B) cliff aversion; (C) surface righting; and (D) forelimb grasp. The individual strain performance is color coded. The different strains of mice from which data were collected are shown along the *x*‐axis where dots represent significant difference between strains determined by a post hoc analysis.

The offspring of all strains exhibited sensorimotor reflexes within the average timeline as specified by Fox [16] and Heyser et al. [23], indicating that all strains of mice had typical neurological development. Although all strains displayed sensorimotor reflexes within the typical age range, there were significant strain differences, measured by average performance across the 15 days of testing for each strain: negative geotaxis [*F* (13, 274) = 11.154, *p* < 0.001], cliff aversion [*F* (13, 274) = 12.785, *p* < 0.001], surface righting [*F* (13, 274) = 8.805, *p* < 0.001], and forelimb grasp [*F* (13, 274) = 5.532, *p* < 0.001] (Figure 2; Table 3). For negative geotaxis, a significant difference was observed between B6 and DBA parental strains between and throughout all days (Figure 2a). Similarly, in cliff aversion, each strain displayed variability in the time taken to turn away from the edge to the secure side. These differences persisted for the entire 15 days of testing, with B6 and BXD86 having a statistically significant difference in time to observe the reflex (Figure 2b). Different from negative geotaxis and cliff aversion, surface righting showed less variability in time taken to restore a pup's position from dorsal to its normal prone position. In forelimb grasp, strains displayed less variability in time to hold the rod and remain suspended above platform, with no significant difference between strains.

**TABLE 3:** Summary of behavioral tests and statistical analysis.

Test	Phenotype	Parameter	ANOVA or ANCOVA (all *p* < 0.001)
Fox Neuro‐developmental Battery	Nervous system maturation	Negative geotaxis	*F* (13, 274) = 11.154
		Cliff aversion	*F* (13, 274) = 12.785
		Surface righting	*F* (13, 274) = 8.805
		Forelimb grasp	*F* (13, 274) = 5.532
Open field	Locomotor activity	Total distance traveled	*F* (13, 280) = 4.54
		Time spent in the center	*F* (13, 280) = 10.54
		Time spent in the periphery	*F* (13, 269) = 34.797
		Time spent moving	*F* (13, 274) = 15.248
		Time spent not moving	*F* (13, 280) = 20.30
		Velocity	*F* (13, 280) = 3.47
Rotarod	Motor coordination, balance	Baseline performance; improvement	*F* (13, 280) = 5.380 *F* (13, 280) = 4.833
		Body speed	*F* (13, 236) = 10.124
		Leg combination	*F* (13, 236) = 15.003
		Stance duration	*F* (13, 236) = 23.536
Gait analysis	Motor coordination, postural control	Swing duration	*F* (13, 236) = 15.737
		Posterior extreme position	*F* (13, 236) = 10.737
		Step cycle	*F* (13, 236) = 23.536
		Duty factor	*F* (13, 236) = 13.934

If we examine development of reflexes over time, we observe significant interaction effects between strain and day for negative geotaxis and cliff aversion, but not for forelimb grasp and surface righting. There are significant differences in the rate of development of these two reflexes (shown in Figure S1).

### 3.2 Motor Activity

#### 3.2.1 Open Field

**FIGURE 3:**
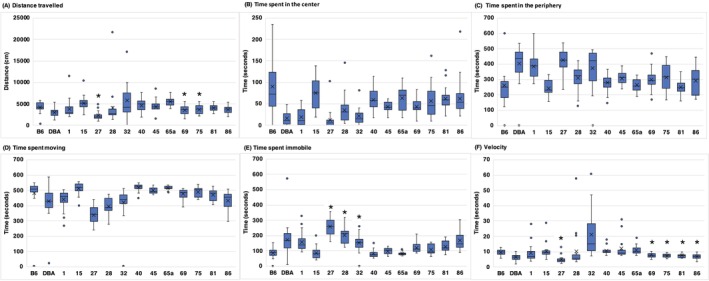
Graphs illustrating strain performance in open field parameters: (A) distance traveled; (B) time spent in the center; (C) time spent in the periphery; (D) time spent moving; (E) time spent immobile; and (F) velocity. The *x* in each box indicates the mean and the bottom line of the box depicts the median. The different strains of mice from which data were collected are shown along the *x*‐axis. The asterisks (*) indicate significance in post hoc tests [dots associated with each strain's behavior are outliers; asterisks (*) indicate significant datapoints].

An open field test was performed as an indicator of spontaneous locomotor activity. The total distance traveled, amount of time spent in the center versus the periphery, time spent moving versus not moving, and the velocity were analyzed (Figure 3). There were significant strain differences for total distance traveled [*F* (13, 280) = 4.54, *p* ≤ 0.001], time spent in the center [*F* (13, 280) = 10.54, *p* ≤ 0.001], time spent not moving [*F* (13, 280) = 20.30, *p* ≤ 0.001], and velocity [*F* (13, 280) = 3.47, *p* ≤ 0.001] over the 10‐min testing period. Figure 3e shows that BXD27, BXD28, and BXD32 spent the least amount of time moving. BXD27, BXD69, BXD75, BXD81, and BXD86 had the lowest velocity when they were moving. When considering the distance traveled within the 10 min of testing, BXD27, BXD69, and BXD75 logged the least distance. No sex differences or significant correlations were found for adult weight and any of the open field parameters (all *p* values > 0.05).

### 3.3 Motor Coordination

#### 3.3.1 Rotarod

To investigate motor coordination, the rotarod test was utilized with a constant speed task. We determined rotarod performance by measuring the latency to fall from the rotarod. A one‐way ANCOVA revealed a significant effect of BXD strain on rotarod performance, particularly on the first day of testing (see Table 3); [*F* (13, 280) = 5.380, *p* < 0.001]. Subsequently, a two‐way ANCOVA showed a significant interaction between BXD strain and day of testing [*F* (13, 280) = 4.833, *p* < 0.001]. Specifically, BXD28, BXD32, BXD65a, BXD69, BXD75, and BXD86 performed below average on the first day of testing. Of the above identified strains, BXD69 and BXD86 had the lowest latency to fall.

**FIGURE 4:**
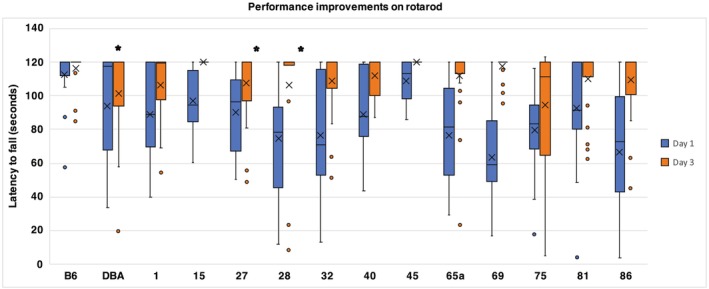
Strain performance in coordination as measured by rotarod. Blue bars represent Day 1 strain performance in latency to fall; orange bars represent Day 3 strain performance in latency to fall. The *x* in the box depicts the mean and the bottom line of the box depicts the median. The different strains of mice from which data were collected are shown along the *x*‐axis. The task is set for 2‐min duration, allowing for optimal timing to assess motor coordination, balance, proprioception, and motor planning variables [asterisk (*) indicates significant datapoints].

To calculate motor performance improvements, the rotarod performance on Day 3 was subtracted from the performance on Day 1 (mean of Day 3 minus mean of Day 1). This allowed us to determine whether previous experience and practice from Days 1 and 2 influenced performance on the last day of testing, indicating motor improvements as the mice became more adept on the rotarod. There were main effects of BXD strain on motor learning [*F* (13, 280) = 4.833, *p* ≤ 0.001]. Figure 4 illustrates the performance improvements and the percentage of improvement from Day 1 to Day 3. This figure shows that DBA was the only strain without a statistically significant improvement from Day 1 to Day 3. Although, BXD27 and BXD 28, along with the parental line DBA show the lowest relative improvement, they do show a statistically significant improvement from Day 1 to Day 3 (mean difference: 7.29 s). Although other strains, including BXD32, BXD65a, BXD69, and BXD86 initially performed more poorly, they had greater motor improvements from Day 1 to Day 3. There was no correlation between adult body weight and motor learning and no sex differences were observed on rotarod performance (*p* > 0.05).

#### 3.3.2 Gait Analysis

**FIGURE 5:**
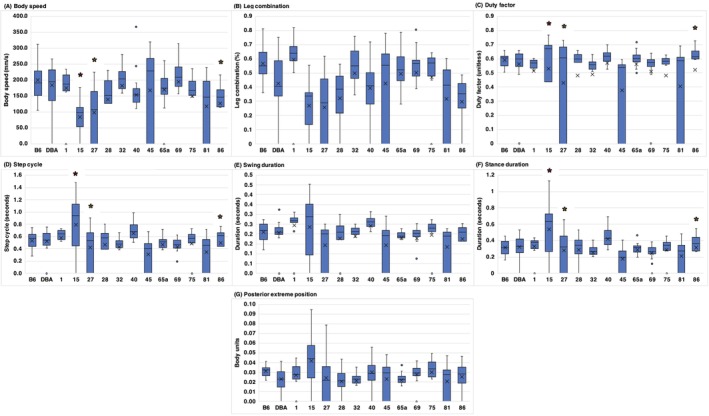
Graphs illustrating strain performance in gait parameters. (A) body speed; (B) leg combination; (C) duty factor; (D) step cycle; (E) swing duration; (F) stance duration; and (G) posterior extreme position. The *x* in the box depicts the mean and the bottom line of the box depicts the median. The different strains of mice from which data were collected are shown along the *x*‐axis [asterisk (*) indicates significant datapoints].

To evaluate motor coordination, specifically postural control, gait analysis was performed. The performance in gait was determined by measuring body speed, leg combination, duty factor, step cycle, swing duration, stance duration, and posterior exterior position. To detect between‐strain differences in gait parameters, an analysis of covariance (ANCOVA) was performed, with body weight and sex as covariates. Statistically significant differences among the strains were identified for all of the parameters measured: body speed [(*F* (13,236) = 10.124, *p* < 0.001)], leg combination [(*F* (13,236) = 15.003, *p* < 0.001)], stance duration [(*F* (13,236) = 23.536, *p* < 0.001)], swing duration [(*F* (13,236) = 15.737, *p* < 0.001)], posterior exterior position (i.e., position of the leg relative to the end of stance phase) [(*F* (13,236) = 10.737, *p* < 0.001)], step cycle [(*F* (13,236) = 23.536, *p* < 0.001)], and duty factor (i.e., proportion of the step cycle where the leg is in contact with the ground) [(*F* (13,236) = 13.934, *p* < 0.001)] (Table 3). Post hoc tests for all gait parameters revealed BXD15, BXD27, and BXD86 displayed the most variable performance on all gait parameters. These strains had lower body speed and stance duration with a longer step cycle and duty factor (Figure 5).

### 3.4 Heritability

For each of 35 phenotypes, heritability (*h* ^2^) was calculated (Table 4). Phenotypes with high heritability have a large effect of genometype on the phenotypic variation. Body weight, as expected, was highly heritable, at *h* ^2^ 0.43–0.55, providing reassurance of correct strain assignment. We had greater power to identify QTL for traits with high heritability, since power to detect QTL is dependent upon heritability, locus effect size, number of genometypes examined, and number of biological replicates. We therefore expected to be more likely to detect QTL for phenotypes such as step cycle (*h* ^2^ = 0.57), stance duration (*h* ^2^ = 0.57), time not moving (*h* ^2^ = 0.49), swing duration (*h* ^2^ = 0.46), leg combination (*h* ^2^ = 0.45), and duty factor (*h* ^2^ = 0.43). We note that four of these five are gait phenotypes, suggesting that aspects of gait are strongly influenced by genometype in the BXD family. At least some of this shared heritability may be due to high correlations between traits (e.g., correlations between step cycle, swing duration and stance duration are *r* ^2^ 0.56–0.96), which would likely result in shared QTL positions.

**TABLE 4:** Heritability (*h* ^2^) of phenotypes.

Phenotype	H2	*p* Value of strain	*n* of individuals measured
Negative geotaxis	0.344352909	8.63E‐19	288
Surface righting	0.302542638	6.36E‐16	288
Cliff aversion	0.379413001	9.77E‐22	288
Forelimb grasp	0.210998721	7.01E‐09	288
Weight	0.429347255	0	289
Weight postnatal day 1	0.488755663	2.97E‐31	285
Weight postnatal day 2	0.45875172	2.79E‐29	285
Weight postnatal day 3	0.548179101	5.93E‐39	288
Weight postnatal day 4	0.508107853	1.41E‐35	288
Weight postnatal day 5	0.524242225	1.79E‐36	287
Weight postnatal day 6	0.517418669	4.22E‐36	288
Weight postnatal day 7	0.518420594	1.15E‐36	288
Weight postnatal day 8	0.52597863	3.43E‐37	288
Weight postnatal day 9	0.495293574	2.00E‐33	288
Weight postnatal day 10	0.489271126	3.88E‐33	288
Weight postnatal day 11	0.45994727	6.59E‐30	288
Weight postnatal day 12	0.456296031	9.34E‐30	288
Weight postnatal day 13	0.435175797	2.03E‐27	288
Weight postnatal day 14	0.434943536	1.86E‐27	288
Weight postnatal day 15	0.436423906	2.12E‐27	288
Distance traveled	0.177181215	3.51E‐07	294
Center time	0.345668111	1.09E‐19	294
Peripheral time	0.317286484	4.66E‐18	294
Time moving	0.445783348	3.62E‐33	294
Time not moving	0.477714731	2.80E‐37	294
Velocity	0.2995408	2.71E‐16	294
Baseline performance	0.238776003	5.82E‐11	291
Performance improvement	0.102956184	0.00270257	291
Body speed	0.364675251	3.26E‐16	250
Leg combination	0.450012444	1.17E‐22	250
Duty factor	0.437611794	6.25E‐22	250
Step cycle	0.578642484	1.94E‐33	250
Stance duration	0.576961367	7.89E‐34	250
Swing duration	0.482718649	2.42E‐23	250
Posterior extreme position	0.3783504	1.92E‐19	250

*Note*: Heritability was calculated as the proportion of total variance explained by strain.

### 3.5 QTL Analyses

A summary of the QTLs is presented below and in Table 5. For each QTL, the effect of each variant was predicted (Supplementary Files 1–15 in Data S1), and these are summarized for each QTL (Table S1). We found three significant QTLs, all from traits associated with gait analysis (Table 5). A total of 13 suggestive QTLs were identified that spanned all of the behavioral tests given. We also mapped males and females independently (Table S2); however, given the reduction in power by using half the individuals, we have concentrated on those QTL from the mean values of all samples.

**TABLE 5:** Summary of QTL analysis of behavioral traits.

GeneNetwork ID	Parameter	Location	View	Position (Mb)	LRS	Marker	Genes	Total n of genes	Threshold	Effect size
20,357	Negative geotaxis			Chr 19: 52.202000	11.13	rs13483669			None	0.346
20,359	Surface righting	Chr 17	40–48	Chr 17: 40.571000	18.28	rs13482981			Suggestive	0.294
20,358	Cliff aversion	Chr 2, 10	116.5–118.9	Chr 2: 116.722000	13.01	rs6381234			Suggestive	0.377
20,360	Forelimb grasp	Chr 10	100.9–104	Chr 10: 102.115000	18.23	rs13480733			Suggestive	0.207
20,362	Distance traveled	Chr 6	63.8–64.8	Chr 6: 63.787000	10.17	rs30759937			Suggestive	0.174
20,365	Time spent in center	Chr 9, 10	81–95	Chr 9: 81.388000	17.65	rs29934845			Suggestive	0.362
20,363	Time spent in periphery	Chr 9, 10	80.5–94.5	Chr 9: 81.388000	12.93	rs29934845			Suggestive	0.623
20,367	Time moving	Chr 9, 18, 10, 15	77.9–81.4; 94.9–99.8	Chr 9: 78.055000	14.57	rs6332933			Suggestive	0.384
20,369	Time not moving	Chr 9, 18, 10, 15	77.9–81.4; 94.9–99.8	Chr 9: 78.055000	14.57	rs6332933			Suggestive	0.481
20,371	Velocity			Chr 6: 114.025000	8.8	rs30499894			None	0.138
20,086	Baseline performance	Chr 5, 6	51.5–52.95	Chr 5: 52.475000	15.91	rs48813171			Suggestive	0.201
20,094	Performance improvement	Chr 12, 4, 1	75–79	Chr 12: 75.295000	14.68	rs29187781			Suggestive	0.185
20,969	Body speed	Chr 8	24.8–42.7	Chr 8: 24.907000	13.76	rs33516211			Suggestive	0.358
21,408	Leg combination	Chr 4	25–32.1	Chr 4: 28.322410	20.77	rs13477622	Fut9, 4930556G01Rik, Map3k7, Fhl5, Gpr63, Ufl1, 1810074P20Rik	7	Significant	0.452
20,967	Duty factor	Chr 19, 16, 4	30–33	Chr 19: 29.979000; Chr 19: 29.981000	18; 15.36	No SNP; rs13483589			Suggestive	0.435
21,427	Step cycle	Chr 3	36.25–37.87	Chr 3: 36.543329	18.5	rs30698864	4930556A17Rik, Gm5148, EG381438, Spry1, 4930594O21Rik, Spata5, Nudt6, Fgf2, Bbs12, Cetn4, Il21, Il2, Adad1, AK029633, AK051829, 4932438A13Rik, Trpc3, Bbs7, Anxa5	19	Significant	0.564
20,971	Stance duration	Chr 3, 10, 4	36.55–37.9	Chr 3: 36.543000	15.81	rs30698864			Suggestive	0.565
20,972	Swing duration			Chr 6: 142.500000; Chr 6: 143.933000	13.06; 12.87	No SNP; rs36263502			None	0.583
21,410	Posterior extreme position	Chr 6	98–105.7; 111–114	Chr 6: 98.309000	15.58	rs6245069	Cntn4, Mitf, Cntn3, 4930587E11Rik, Foxp1, Gm765, Pdzrn3, Ppp4r2, 4930595L18Rik, Cntn6, Rybp, Shq1, Chl1, 6030492E11Rik, Glt8d4, Gxylt2, A930015G24Rik, AK020861, 9130401L11Rik, Eif4e3, 1700049E22Rik, AK134520, EG207157, Gm9871, Grm7, Srgap3, Lmcd1, Setd5, Cpne9, Rad18, Lhfpl4, AK016007, AK016007, Brpf1, Mtmr14, Cidec, Arpc4, Tada3l, Tada3, Camk1, Ttll3, Ogg1, AK138856, Il17rc, Rpusd3, Fancd2, Thumpd3, 5031434C07Rik, Gt(ROSA)26Sor, Irak2, Jagn1, D630042P16Rik, Ssu2, Il17re, Prrt3, 1700054K19Rik, 9530092B13Rik, Cav3, Tmem111, Emc3, 6720456B07Rik, Brk1	62	Significant	0.387

*Note*: The genes that underlie the significant QTL intervals are provided (green: meets significant threshold; orange: suggestive threshold; gray: does not meet significant or suggestive thresholds).

#### 3.5.1 Neurodevelopmental Battery

The genome‐wide QTL analysis did not identify any significant QTLs for any elements of the battery, but suggestive QTLs were noted for righting reflex on Chr 17; for cliff aversion on Chr 2 and Chr 10; and for forelimb grasp on Chr 10 (Figure S2).

#### 3.5.2 Open Field

Genome‐wide QTL mapping for quantified traits in the open field test did not identify any significant QTLs. Several suggestive QTLs were found on Chr 9, Chr 18, Chr 10, and Chr 15 for time spent moving and not moving; on Chr 10 and Chr 9 for time spent in center and periphery; and on Chr 6 for total distance traveled (Figure S3).

#### 3.5.3 Rotarod

The genome‐wide QTL map did not identify any significant QTLs for rotarod measures but identified suggestive QTLs for baseline performance on Chr 5 and Chr 6, and for performance improvement on Chr 12, Chr 4, and Chr 1 (Figure S4).

#### 3.5.4 Gait Analysis

**FIGURE 6:**
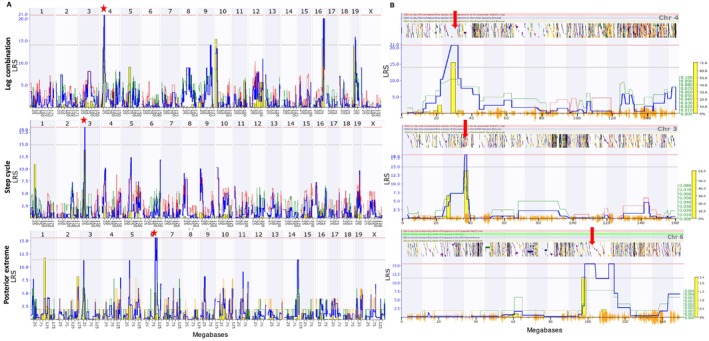
Genome‐wide linkage map of leg combination, step cycle, and posterior extreme position (top to bottom) of gait analysis to determine postural control. The overall blue trace shows the LRS. (A) Genome‐wide QTL map showing a significant QTL on chromosome 4 for leg combination, chromosome 3 for stance duration, and chromosome 6 for posterior extreme position. (B) Interval QTL map of chromosomes 4, 3, and 6 using three test week performance with bootstrap analysis. The lower gray horizontal line represents suggestive LRS genome‐wide threshold at *p* ≤ 0.63. The upper pink horizontal line represents significant LRS genome‐wide threshold at *p* ≤ 0.05. The bottom orange marks indicate SNP density [asterisk (*) indicates significant QTL; red down arrow (↓) indicates significant QTL interval with genes].

The genome‐wide QTL map for gait analysis identified three significant QTLs in different gait parameters (Figure 6). For leg combination, a significant QTL was located on the proximal end of Chr 4 [GeneNetwork Trait ID_21408] and spanned from 25 to 32.1 Mb with a LRS score of 20.77. A significant QTL for step cycle was also mapped to the proximal end of Chr 3 and it spanned from 36.25 to 37.87 Mb with a LRS score of 18.5 [GeneNetwork Trait ID_21427]. A significant QTL for posterior extreme position was mapped to the distal end of Chr 6 [GeneNetwork Trait ID_21,410] and spanned from 98 to 114 Mb with a LRS score of 15.58. Other parameters had suggestive QTLs: body speed on Chr 8; duty factor on Chr 19, Chr 16, and Chr 4; stance duration on Chr 3, Chr 10, and Chr 4; leg combination on Chr 5, 16, and 19; and duty factor on Chr 10, 4, 16, and 19 (Figure S5).

#### 3.5.5 Candidate Genes for Significant QTLs

With the identification of significant QTLs for three traits in gait analysis, our attention focused on the best candidate genes that might underlie each QTL. To this end, we identified 88 genes that were mapped to the three significant QTL regions (Table 5).

As described in Section 2, a series of criteria were employed to prioritize promising candidates based upon: (1) the gene is expressed in central nervous system (CNS) and/or skeletal muscle; (2) the gene is associated with a motor function related to DCD‐like behavior; and (3) presence of nonsynonymous SNPs in coding region of the gene. The expressed sequence tags and Riken clones within the gene list were not evaluated since they are currently non‐annotated in terms of expression pattern and function. Of all the genes observed across three significant QTLs, 59 genes met criterion 1; seven of these 59 genes met criteria 1 and 2 (Table 5); and three of these seven genes met all three criteria (Table S3).

Tissue‐specific expression is an important factor for determining the role of genes in a given disorder. The motor coordination problems are important for a diagnosis of DCD and are likely associated with genes involved in brain development or motor regions and/or skeletal muscle function [24]. Hence, the initial assessment of the 88 candidate genes was examined to determine expression in brain and skeletal muscle tissues relative to other tissues using NCBI and MGI databases. Of the 88, we identified 59 genes that had higher expression in CNS and skeletal muscles (Table S3). Secondly, the functional role of 59 genes was explored using NCBI resource and identified the following genes: *Gpr63, Spata5, Trpc3, Cntn6, Chl1, Grm7*, and *Ogg1* as meeting criterion 2. Lastly, the SNP variant browser was used to scan the above seven genes and identified that three of these genes have non‐synonymous polymorphisms. Thus, three candidate genes, *Spata5, Cntn6*, and *Chl1*, emerged from the two significant QTL regions that met all three criteria and were therefore considered as priority genes to explore in relation to the clinical presentation of DCD.

## 4 Discussion

The results of this study show that there is significant variability in posture, balance, motor activity, and sensorimotor coordination amongst the BXD RI strains as measured by gait analysis, rotarod, and open field behavioral assays; this bodes well for using the BXD strains to explore the structural, functional, and genetic aspects of motor behavior. To our knowledge, this is the first study to explore the motor variability in BXD strains of mice in order to explore DCD‐like phenotypes and their genetic underpinnings. Of note is that particular strains of BXD mice share core symptoms displayed by individuals with DCD. For example, BXD75 and BXD86 have motor coordination difficulties that are not unlike individuals with DCD who have poor static and dynamic balance [25, 26, 27], decreased spontaneous locomotion [28, 29, 30, 31, 32], and altered gait patterns [33, 34, 35]. As seen in the BXD15, BXD27, BXD28, BXD75, and BXD86 strains of mice, children with DCD also struggle to improve their motor performance, even with increased opportunity to practice. Individuals with DCD continue to show variable and less accurate motor performance over time, which is also seen in some strains of BXD mice. The observed gait differences across BXD strains are also consistent with gait patterns of children with DCD, who show abnormal walking patterns [36], including bilateral asymmetry [33, 34, 37], decreased ability to coordinate lower limbs [33, 37], greater step width [35], and longer time in stance phase [35]. Thus, the behavioral phenotypes seen in this study are similar to what may be seen in individuals with DCD and have the potential to offer insight into the genetic and neuroanatomical underpinnings of motor coordination impairments. At first blush, these results show great potential for a murine model to study the etiology of DCD.

### 4.1 Hypothesis‐Driven Selection of Genotypes and Phenotypes

Generally, testing a small number of strains in a reference panel yields fewer behavioral differences and a diminished probability for detecting QTLs; however, in this study, we saw significant inter‐strain variability for all traits studied as well as the detection of significant QTLs. These findings would seem likely due to the hypothesis‐driven selection of BXD strains to study, along with having a population with large inherent differences as embodied in B6 vs. DBA and the BXD strains that emerge from the recombinant inbred cross. Our results were undergirded by the extensive phenotyping that has been done with these mice, which provided many selection criteria from which to choose. One selection criterion was based on the observed motor phenotypes in BXD strains of mice. As such, strains that showed variable performance on motor coordination tasks were chosen, along with those who had variation in brain structures that are associated with motor skills.

While some strains were selected based on known brain structural differences, others were selected based on the variability in motor performance in posture, balance, and locomotor activity tasks. Thus, BXD75 and BXD86 were amongst the strains that were chosen based on motor performance. Similar to previous findings, we also observed BXD75 and BXD86 to struggle with motor coordination and balance [13, 14]. On the open field test, Brigman et al. [14] and Philip et al. [13] found that BXD75 and BXD86 presented with sedentary behavior and below average general locomotion, which may be due to motor coordination impairments. While the reason for difficulties in general locomotion and balance for both BXD75 and BXD86 may not be clear, other studies identify why these strains may struggle with motor coordination in general. Loos et al. [38] and Laughlin et al. [39] evaluated strain differences in attentional performance in a serial reaction time task and an operant learning task, respectively. Loos et al. [38] found that BXD75 struggled with attentional performance, while Laughlin et al. [39] found that BXD86 had difficulty with behavioral flexibility in order to change or stop a response.

It is known that attention and behavioral flexibility are needed for motor coordination of functional motor tasks [38, 39]. Impaired attentional abilities and lack of ability to change or stop a response, may both depend on neuroanatomical differences. In this vein, Jan et al. [40] and Gaglani et al. [41] found that BXD75 and BXD86 both have lower cerebral cortex volume, particularly somatosensory cortex volumes. The somatosensory cortex plays a critical role in processing afferent somatosensory input and contributes to the integration of sensory and motor signals that are necessary for skilled movement; abnormalities in this structure may lead to motor dysfunction [40]. Therefore, it is plausible to suggest that structural differences in the somatosensory cortex may be contributing to attentional and behavioral inflexibility in BXD75 and BXD86 that lead to poor motor coordination and balance.

A second selection criterion was based upon neuroanatomical phenotypes, which are important to assess in neurodevelopmental disorders, such as DCD, as anatomy underlies behavior [42]. As such, this presented another criterion for selection and our initial selection criteria applied to BXD strains, that is, mining the published data on cerebellar volume—a structure that is intimately related to motor coordination. In fact, abnormalities in cerebellar structure and functions are hypothesized and shown to be involved in many neurodevelopmental disorders, including DCD [15, 43]. Badea, Johnson, and Williams [44] compared various brain structures in BXD strains of mice and found that BXD27 had the lowest total cerebellar volume, whereas BXD15 and BXD28 had moderate cerebellar volume and BXD1 had the highest cerebellar volume when compared to each other and to the parental strains. As the cerebellum is involved in motor coordination and learning [45], the strains with smaller cerebellar volumes (BXD15, BXD27, and BXD28) show substantial alterations in motor coordination and balance as seen in the rotarod, open field, and gait tasks. On the other hand, BXD1 mice have the largest cerebellar volume, comparable to B6, performed well on all motor coordination tasks and had a typical gait pattern. Our findings are further strengthened by Ceccarelli et al. [46] who studied the effects of altered cerebellar development on motor coordination and balance in mice. They found that gene knockout of *Tsi21/Btg2* in the Btg family of mice caused altered migration of cerebellar granule precursors causing abnormal cerebellar volume and thickness, which led to motor coordination and balance impairments, as seen on the rotarod. Further, Galante et al. [47] found that abnormal cerebellar structure, consisting of reduced cerebellar neuronal number and consequently reduced neuronal density in the cerebellum of Tc1 mice led to major deficits in motor coordination and balance on the rotarod, decreased motor performance improvements on consecutive days of testing on the rotarod, reduced ability to habituate to the environment in the open field, and differences in gait patterns [47]. Vinueza Veloz et al. [48] also found that mice with abnormal cerebellar structure with lack of Purkinje cell output exhibited short and irregular strides recorded by footprint analysis, had difficulties with keeping balance on the rotarod, and displayed reduced open field locomotion activity.

### 4.2 Motor Behavior QTLs

Despite the limited number of BXD strains used in this analysis, we were able to detect three significant QTLs. This may be due to a number of features of the present work, including fortuitous outcomes, the so‐called Winner's Curse [49]. The pre‐selection of strains for testing (see above) is likely to provide a more robust filter to enable stronger correlations between behavior and the genome—that is, strains at the poles of the distribution were chosen, producing a larger effect size (see right‐most column in Table 5). In addition, the large number of mice used in each group reduced environmental variation, allowing a more accurate estimate of strain mean for each phenotype, and therefore increased power to identify QTLs. We can be slightly reassured by the fact that our most significant QTL also had high heritability, supporting the strong genetic effect on these traits.

The three genes, *Cntn6*, *Chl1*, and *Spata5*, that passed all three criteria proved to be very interesting candidates. *Cntn6* and *Chl1* genes emerged from the posterior extreme position parameter. Genetic mutation of *Chl1* in mice have shown that partial or complete deletion leads to cognitive, behavioral, motor, and coordination deficits [50, 51]. *Cntn6*, a member of the immunoglobulin superfamily is expressed exclusively nervous system development. Behavioral studies have shown that Cntn6‐deficient mice displayed impaired motor coordination [52]. We also identified *Spata5* from the step cycle gait parameter. *Spata5* is spermatogenesis‐related gene and a member of the ATPase family. They are abundantly expressed throughout the brain and mutations of the gene in the human population have been found to result in major perturbations in brain development [53]. The details of *Spata5* in various cellular processes, particularly in brain development is still to be defined.

### 4.3 Genes, Gait Behavior, and Previous QTL Analyses

There is an indication that DCD has an underlying genetic component due to its high heritability (~70%) [54, 55]. Evidence suggests familial clustering of DCD [56], as well as an increased rate of copy number variations [57]. To explore such gene‐phenotype correlations in past work on motor control, we examined previously acquired mouse phenotypic data in MGI. Here we find that the chromosomal location of the gait QTLs overlapped with abnormal phenotypes that included emotionality, stress [58], anxiety [59], motor coordination [60], and locomotor activity [61]. These phenotypes are provocatively present in many children with DCD who experience stress, particularly in physical education classes [62]. Sometimes, these children have difficulty with behavioral and emotional responses [63] and show more aggression during play than their same‐aged peers [64], including unprovoked hitting, kicking, and grabbing [65]. Problems collectively experienced in the physical, cognitive, emotional, and social domains of development could limit the skills and resources from which children with DCD can draw to cope adaptively with stressful situations. Persistent problems experienced in this context will negatively impact children's functioning and well‐being [66]. Overall, the investigation of previously reported studies within significant mapped QTL identified overlapping loci for other annotated abnormal phenotypes that may be associated with motor‐related phenotypes.

The suggestive QTLs draw attention to loci that may be worth tracking and screening of rare variants. Suggestive QTLs were mapped to various chromosomal regions, and they overlapped with various phenotypes in MGI using the “phenotype, alleles, and disease models” query. Thus, for gait parameters [(Chr 8: 24.8–42.7 Mb); (Chr 19: 30–33 Mb); (Chr 3: 36.55–37.9 Mb)]—body speed, duty factor, and stance duration—the query output generated various phenotypes including ethanol consumption [67], motor coordination [52, 60] and learning and memory [68]. For rotarod parameters [Chr 5 (51.5–52.95 Mb) and Chr 12 (75–79 Mb)]—baseline performance and performance improvement—the query output generated various phenotypes including weight control [69], motor coordination [70], locomotor activity [71], organ weights, and limb lengths [72]. When we queried the suggestive QTLs for open field parameters—distance traveled, time spent in center, time spent in periphery, time moving, and time not moving—[(Chr 6: 63.8–64.8 Mb); (Chr 9: 81–95 Mb); (Chr 9: 80.5–94.5 Mb); (Chr 9: 77.9–81.4 and 94.9–99.8 Mb)], the query output generated phenotypes that included locomotor activity [71], mood [73], learning and memory [74], cerebellar development [75], and anxiety‐like behavior [76]. Finally, we input the suggestive QTL location for the neurodevelopmental battery [(Chr 15: 40–48 Mb), (Chr 2: 116.5–118.9 Mb), and (Chr 10: 100.9–104 Mb)] the query output generated anxiety [77], body weight [78], locomotion activity [79], feeding [80], and circadian rhythms [81].

In summary, the rich genetic and phenotypic diversity in the BXD RI strains and analysis of behavioral traits allowed us to identify three significant chromosomal regions that correlate with aspects of gait, which is known to be atypical in children with DCD in terms of timing, coordination, and symmetry [33, 34, 35]. Using bioinformatic resources, we identified three candidate genes in these QTL regions (*Cntn6, Chl1*, and *Spata5*). In addition, overlapping loci with abnormal phenotypes (e.g., anxiety, motor coordination, locomotion activity) were observed at some of the significant mapped regions. The most promising candidate genes within the QTLs contain nonsynonymous sequence polymorphisms that may be involved in the regulation of motor phenotypes. To date, no connections have been found between these candidate genes and DCD‐related etiology. Therefore, in the future, it will be of interest to determine if these molecules play a role in DCD phenotype regulation by performing genetic‐association and functional studies that help to illuminate the etiology of DCD.

An important aspect of this work is the identification that different DCD‐related phenotypes segregate in the BXD population—that is, strains can be poor on some tasks, while performing well on others. This allows us to genetically dissect different DCD‐related phenotypes and will allow us to identify genes underlying specific aspects of the disorder. This is important, as this can be used to inform findings from human studies of DCD; comparing the human work with animal studies, we will be able to identify which specific phenotypes within the disorder the gene contributes. This promises to be an informative path as indicated by a recent human GWAS report that has started to identify genes associated with DCD [8].

### 4.4 Concluding Remarks

The replication of these studies should be conducted in additional BXD strains to corroborate the preliminary QTLs identified here, and to explore variation in motor coordination and corresponding QTLs. Although we estimated high heritability values for many of our traits, these only translated into a small number of significant QTLs. There are two primary reasons for this. First, we used a relatively small number of strains, but with high within‐strain replication. This allowed us to get accurate estimates of strain means and SD, but power to detect QTLs is increased by increasing the number of genotypes tested (i.e., increasing the number of strains). Second, it is likely that these traits are influenced by many genetic loci. As more loci contribute to a trait, the effect size of any particular QTL decreases, reducing power to detect them. That is, if all heritable variation is explained by a single QTL (a locus effect size of 1), it is much easier to map than if the same amount of heritable variation is explained by 20, equal effect size QTL (each with a locus effect size of 0.05). Again, increasing the number of strains used would increase our power.

As a companion paper to this study, we address the variation in motor learning and corresponding QTLs and assess whether we can access phenotypes and genotypes relevant to DCD [82]. Additionally, brain structural variability will be explored of BXD strains of mice to correlate the structural and behavioral differences seen in these 12 strains of mice. These combined studies should provide important insights into our understanding of the etiology and genetics of DCD.

## Conflicts of Interest

The authors declare no conflicts of interest.

## Supporting information


**Figure S1.** Assessments of strain‐by‐day effect in BXD mice in the Fox Neurodevelopmental Battery. The datapoints for the strains (y‐axis) for each mouse (the colored circles indicate the mean at each day) and the x‐axis is the score/performance/value is shown for (A) negative geotaxis; (B) cliff aversion; (C) surface righting; and (D) forelimb grasp. The days of testing (1–15) are indicated by the color coding bar to the right of each parameter. There is typically an improvement of time (darkest blue to lightest blue) in each reflex. A significant interaction effect is shown for negative geotaxis (*p* = 3.9e‐05; with a significant post hoc difference due to the B6 vs. DBA, B6 vs. BXD75, and BXD27 vs. DBA comparisons). A signficant interaction effect is also seen in cliff aversion (*p* = 0.000179; with a significant post hoc differences due to: B6 vs. BXDs 86, 45, 15, 1, 81, 69, 75, 40, 65a, and BXD32 vs. BXD84 and BXD45 comparisons).
**Figure S2.** Genome‐wide linkage map of negative geotaxis, surface righting, cliff aversion, and forelimb grasp on the Fox Neurodevelopmental Battery to determine nervous system maturation. The overall blue trace shows the LRS. The genome‐wide QTL map showing suggestive QTLs on Chromosome 17, 2, 10 and 13 for Fox Neurodevelopmental Battery parameters. No QTL identified in negative geotaxis. The lower gray horizontal line represents suggestive LRS genome‐wide threshold at *p* ≤ 0.63. The upper pink horizontal line represents significant LRS genome‐wide threshold at *p* ≤ 0.05.
**Figure S3.** Genome‐wide linkage map of distance traveled, time spent in the center, time spent in the periphery, time spent moving, time spent not moving, and velocity of open field test to determine locomotor activity. The overall blue trace shows the LRS. The genome‐wide QTL map showing suggestive QTLs on Chromosome 6, 9, 10, 15 and 18 on open field parameters. No QTL identified in velocity parameter. The lower gray horizontal line represents suggestive LRS genome‐wide threshold at *p* ≤ 0.63. The upper pink horizontal line represents significant LRS genome‐wide threshold at *p* ≤ 0.05.
**Figure S4.** Genome‐wide linkage map of baseline performance and improvements of standard rotarod to determine motor coordination and balance. The overall blue trace shows the LRS. The genome‐wide QTL map showing suggestive QTLs on Chromosome 5, 6, 4 and 12 on standard rotarod. The lower gray horizontal line represents suggestive LRS genome‐wide threshold at *p* ≤ 0.63. The upper pink horizontal line represents significant LRS genome‐wide threshold at *p* ≤ 0.05.
**Figure S5.** Genome‐wide linkage map of body speed, duty factor, stance duration, and swing duration of gait analysis to determine postural control. The overall blue trace shows the LRS. The genome‐wide QTL map showing suggestive QTLs on Chromosome 5, 6, 4 and 12 on standard rotarod. The lower gray horizontal line represents suggestive LRS genome‐wide threshold at *p* ≤ 0.63. The upper pink horizontal line represents significant LRS genome‐wide threshold at *p* ≤ 0.05.

**Tables S1‐S3.** Supporting Information.

## Data Availability

The data that support the findings of this study are openly available at GeneNetwork (https://genenetwork.org/).
