# Supplementary figures and images for: Investigating Motor Coordination Using BXD Recombinant Inbred Mice to Model the Genetic Underpinnings of Developmental Coordination Disorder
Source: Genes Brain Behav. 2025 Mar 12;24(2):e70014. doi: 10.1111/gbb.70014 (PMC11898013; doi:10.1111/gbb.70014)

(A) Negative geotaxis

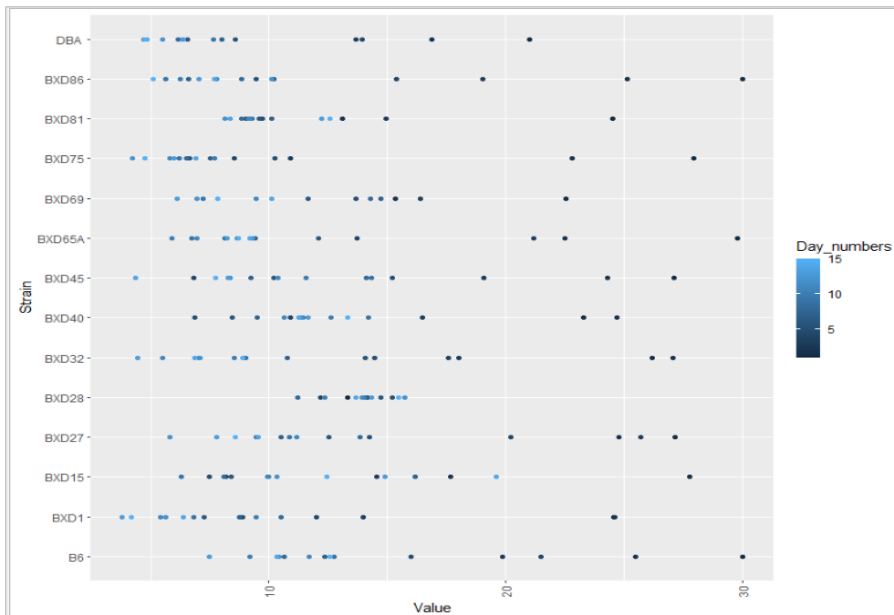

(B) Cliff aversion

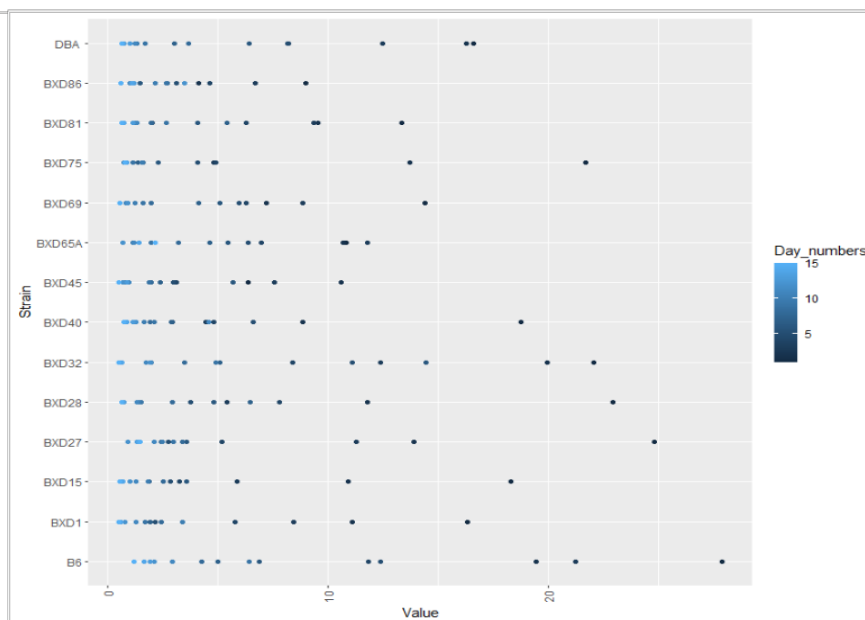

(C) Surface righting

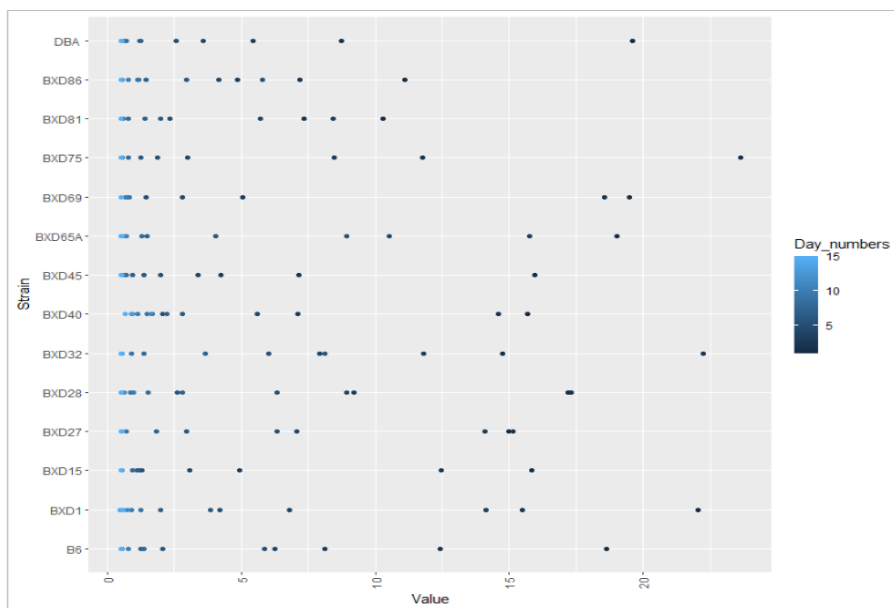

(D) Forelimb grasp

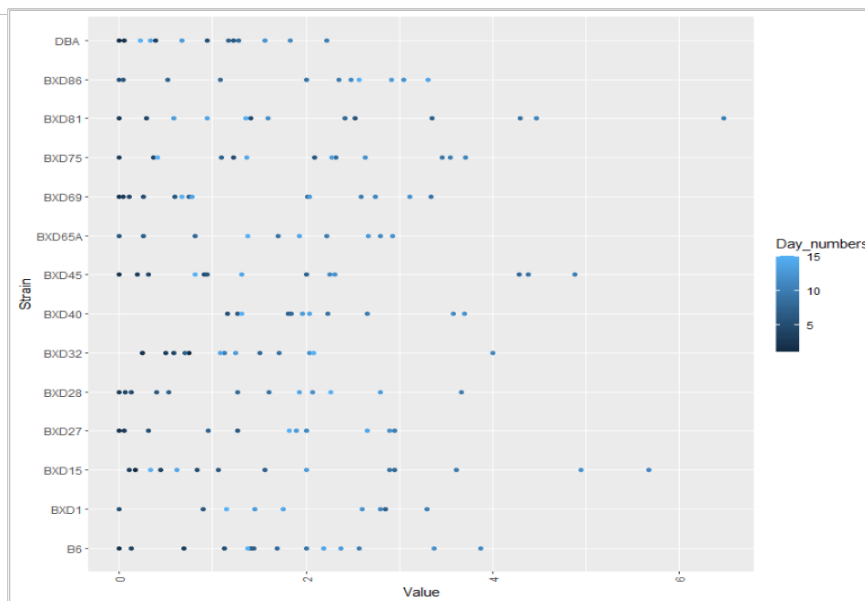

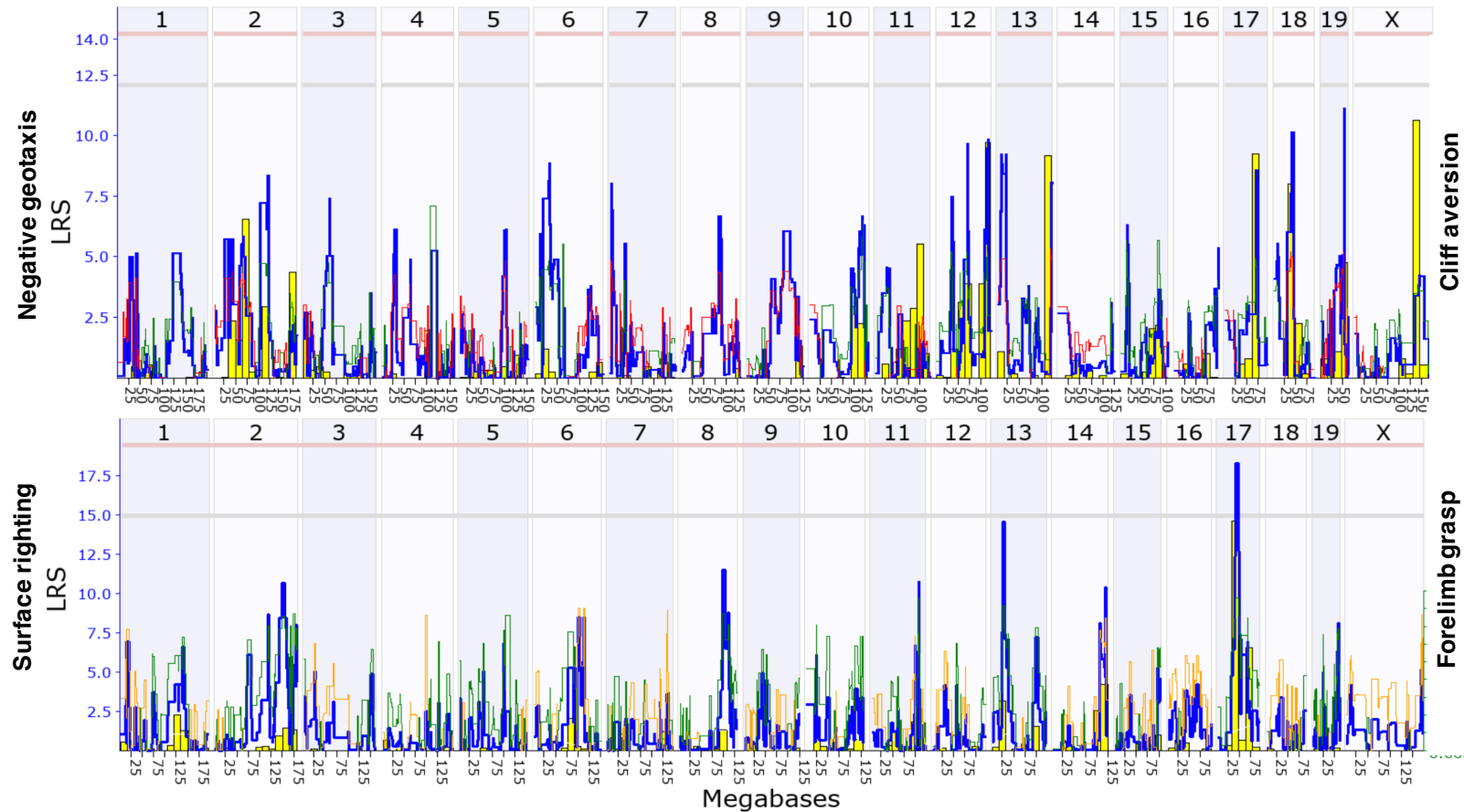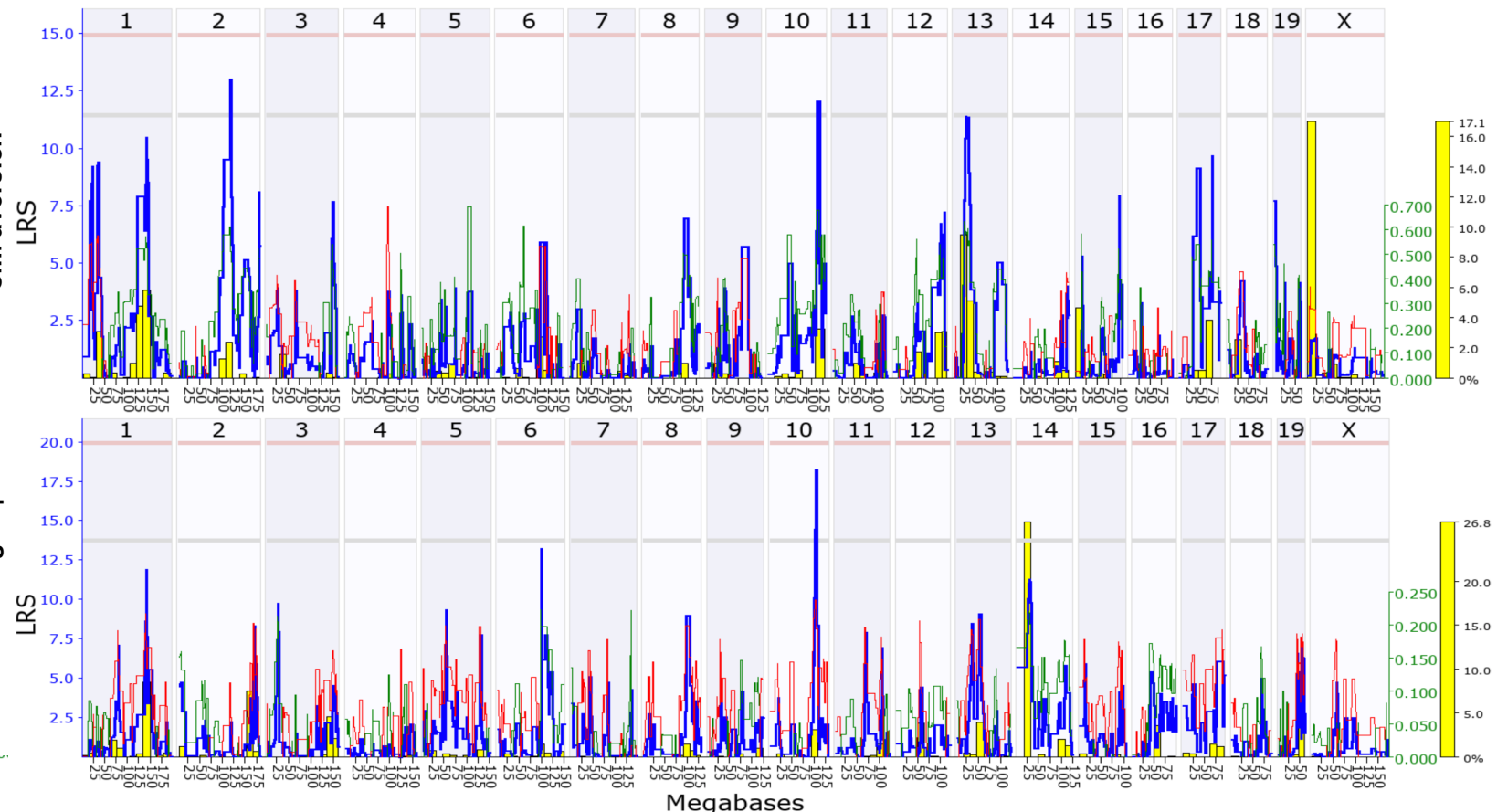

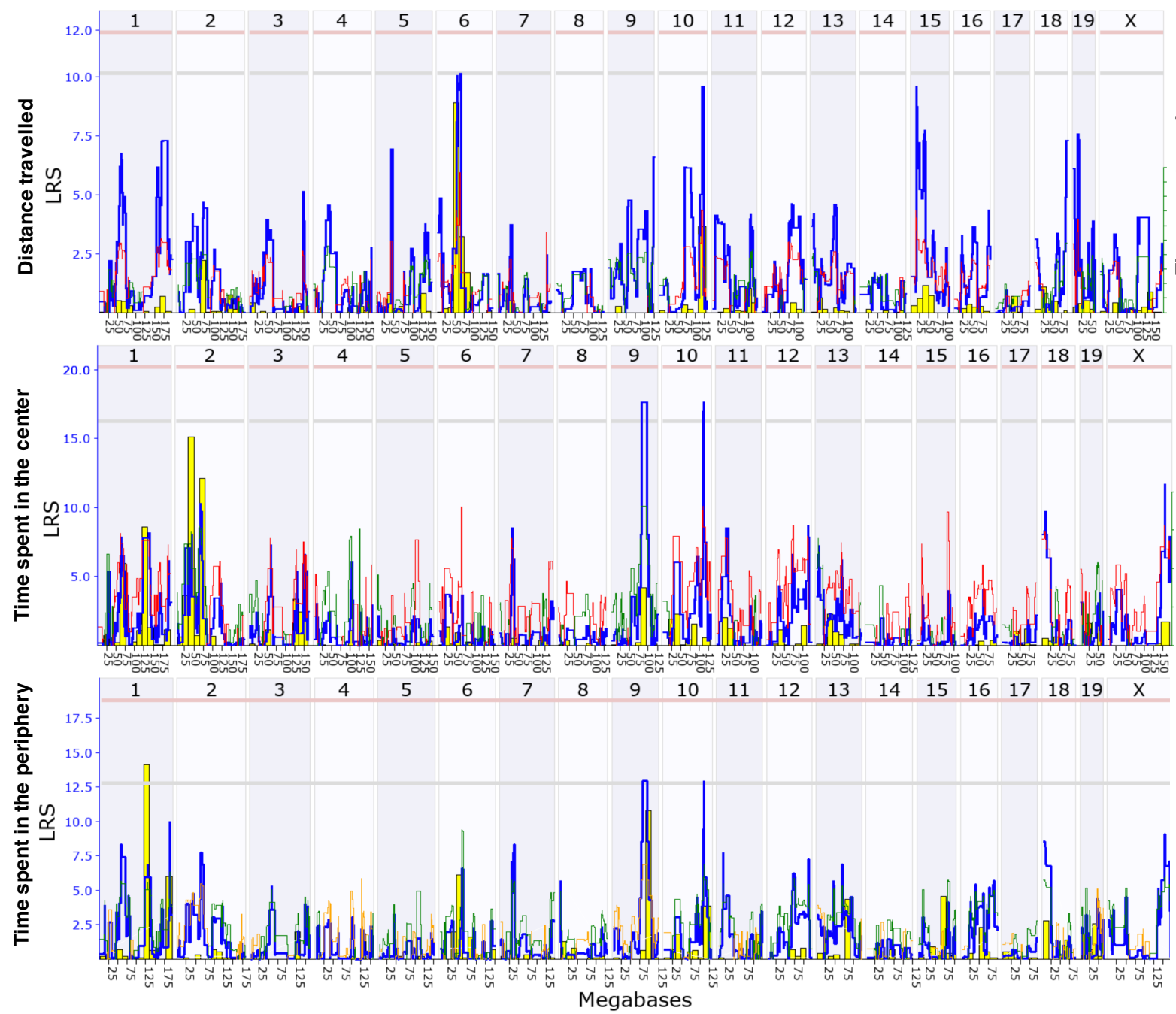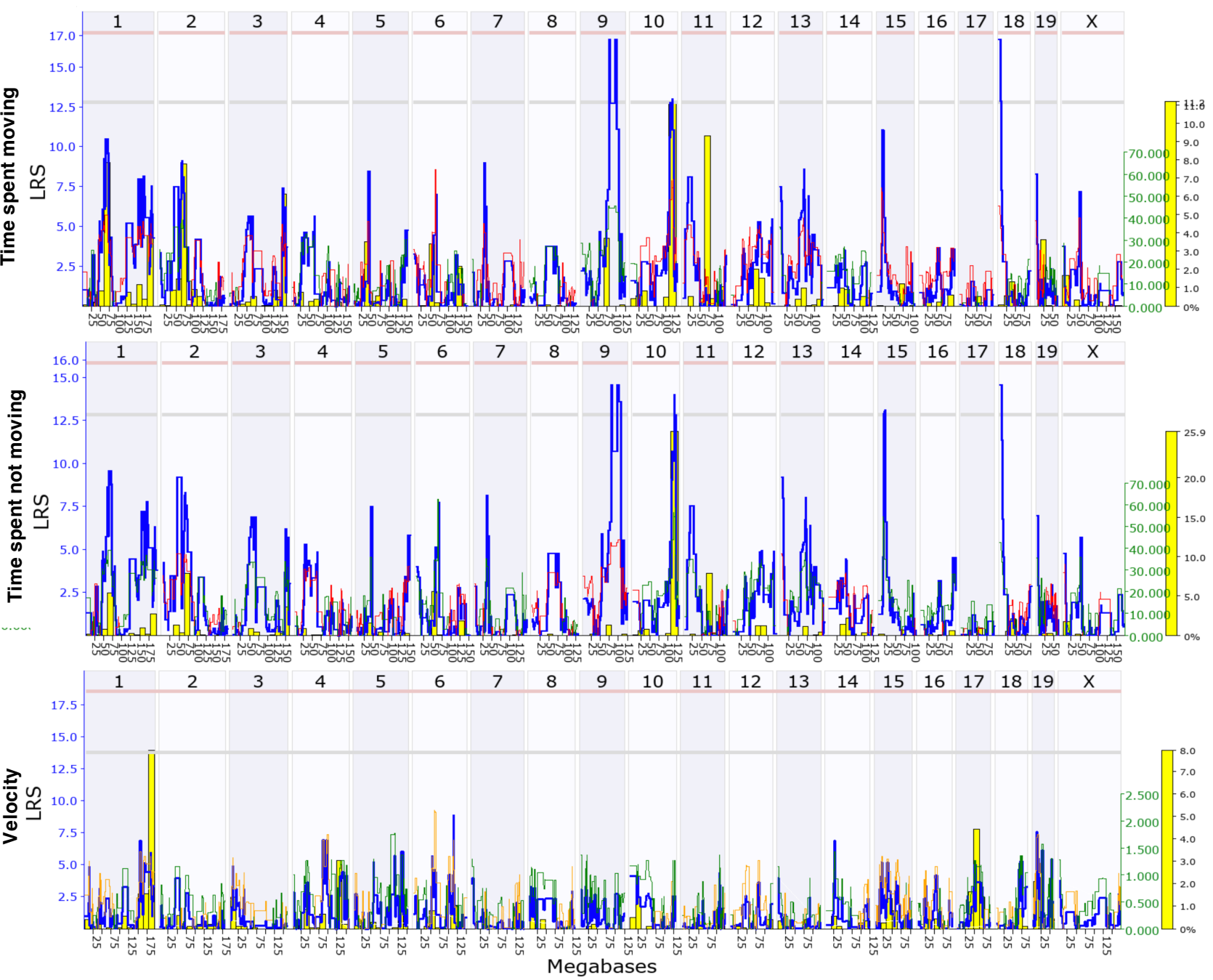

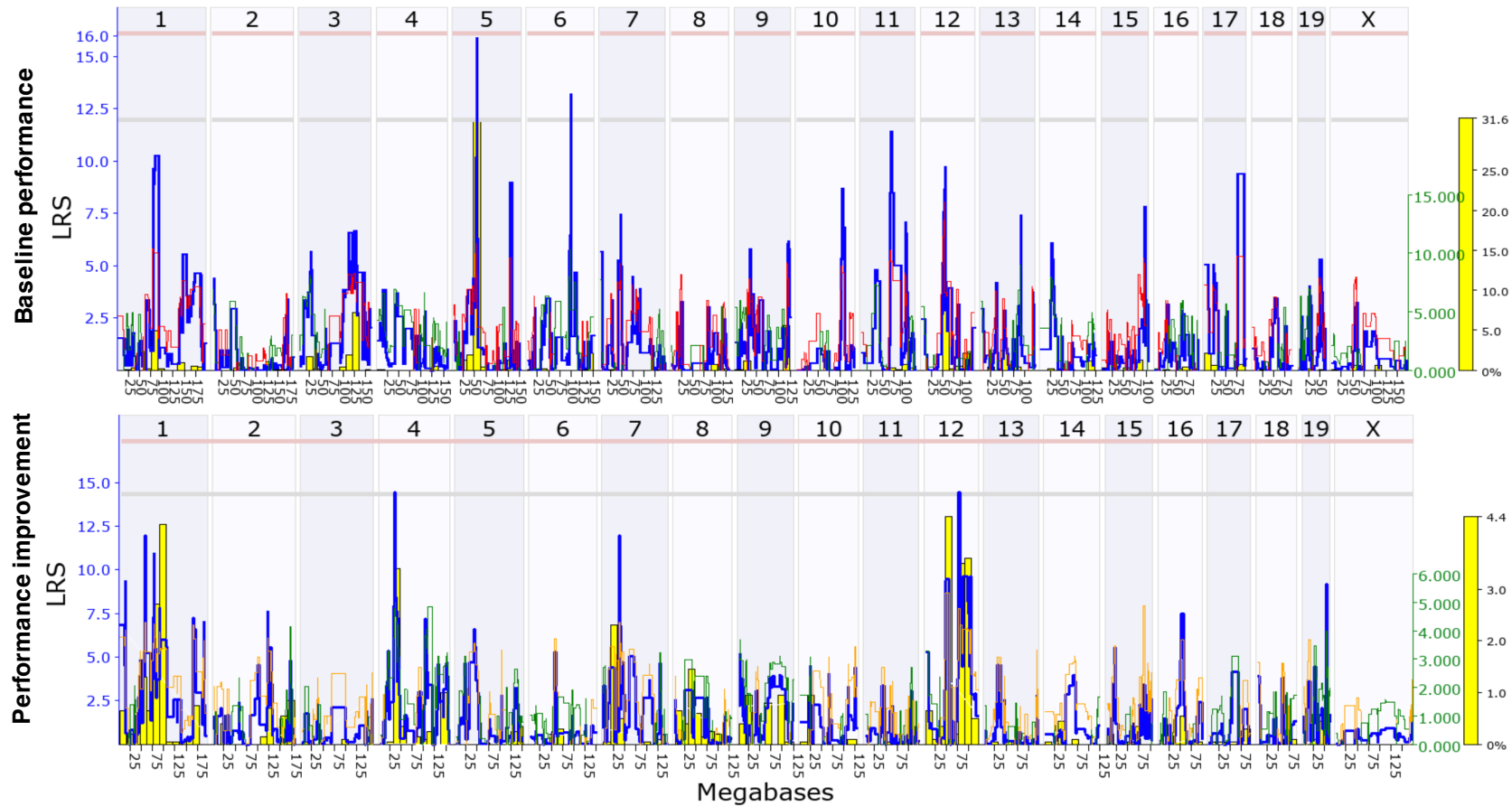

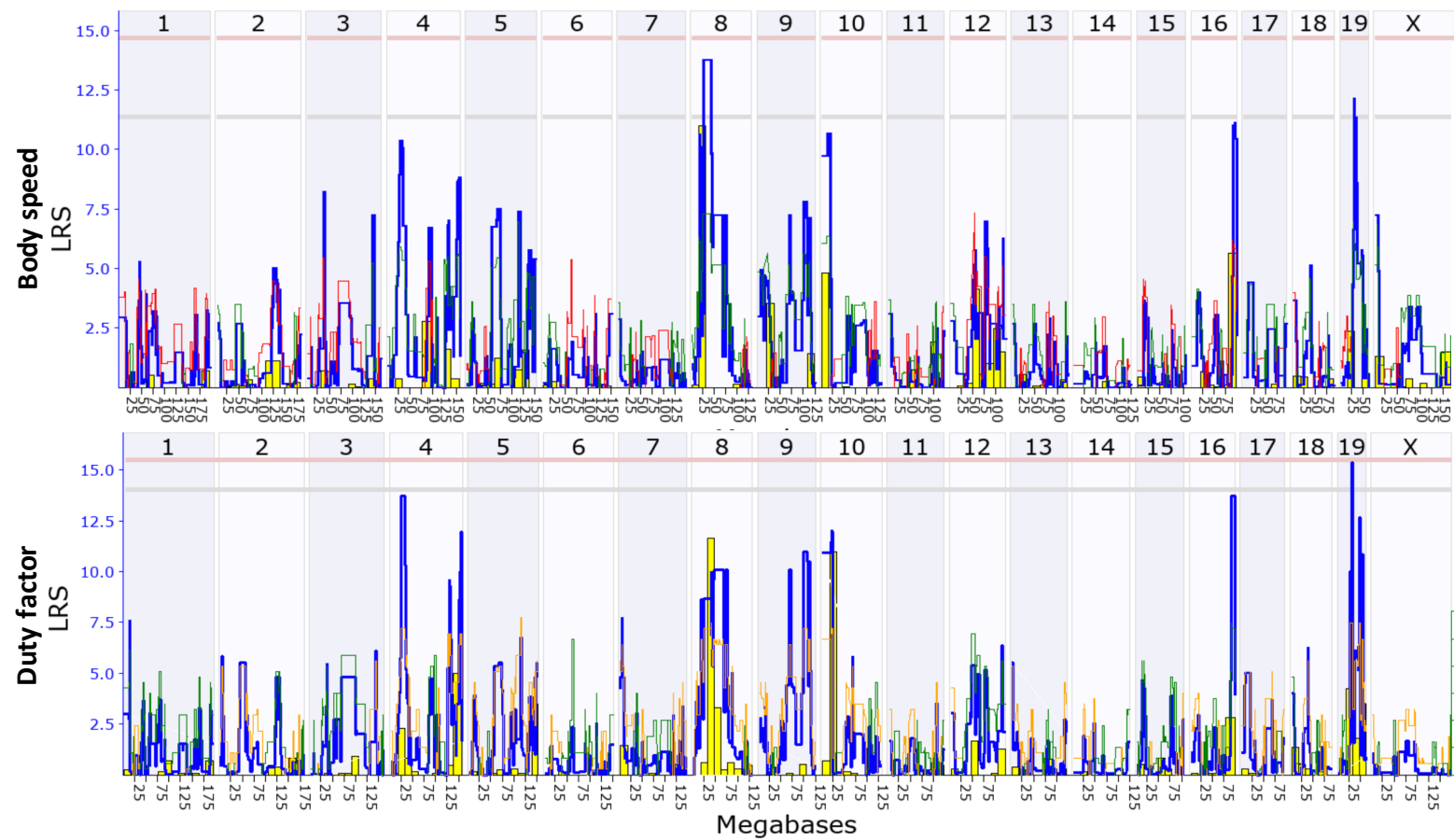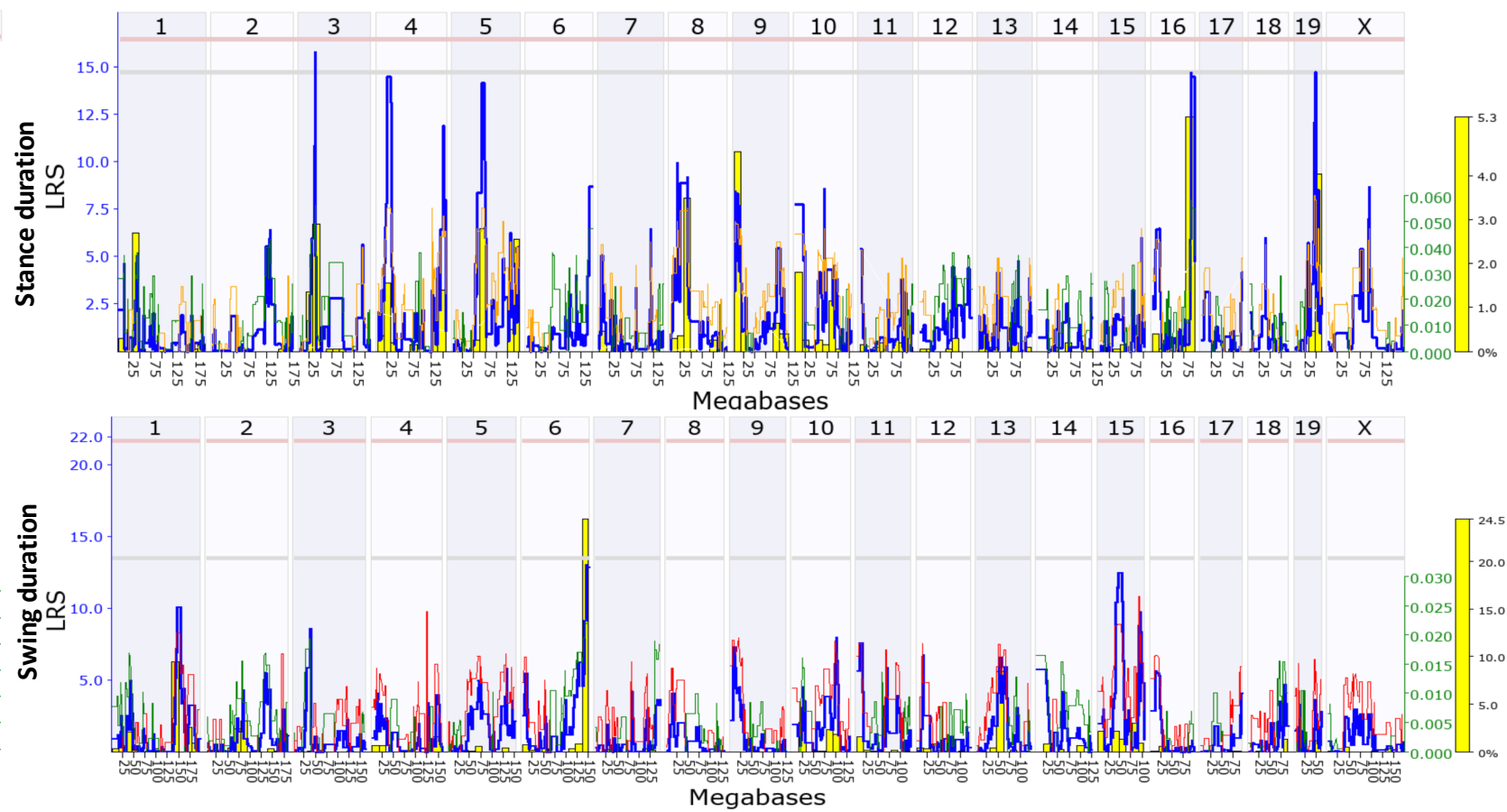

Supplement: Supplementary file 1 — Figure S1. Assessments of strain‐by‐day effect in BXD mice in the Fox Neurodevelopmental Battery. The datapoints for the strains (y‐axis) for each mouse (the colored circles indicate the mean at each day) and the x‐axis is the score/performance/value is shown for (A) negative geotaxis; (B) cliff aversion; (C) surface righting; and (D) forelimb grasp. The days of testing (1–15) are indicated by the color coding bar to the right of each parameter. There is typically an improvement of time (darkest blue to lightest blue) in each reflex. A significant interaction effect is shown for negative geotaxis (p = 3.9e‐05; with a significant post hoc difference due to the B6 vs. DBA, B6 vs. BXD75, and BXD27 vs. DBA comparisons). A signficant interaction effect is also seen in cliff aversion (p = 0.000179; with a significant post hoc differences due to: B6 vs. BXDs 86, 45, 15, 1, 81, 69, 75, 40, 65a, and BXD32 vs. BXD84 and BXD45 comparisons). Figure S2. Genome‐wide linkage map of negative geotaxis, surface righting, cliff aversion, and forelimb grasp on the Fox Neurodevelopmental Battery to determine nervous system maturation. The overall blue trace shows the LRS. The genome‐wide QTL map showing suggestive QTLs on Chromosome 17, 2, 10 and 13 for Fox Neurodevelopmental Battery parameters. No QTL identified in negative geotaxis. The lower gray horizontal line represents suggestive LRS genome‐wide threshold at p ≤ 0.63. The upper pink horizontal line represents significant LRS genome‐wide threshold at p ≤ 0.05. Figure S3. Genome‐wide linkage map of distance traveled, time spent in the center, time spent in the periphery, time spent moving, time spent not moving, and velocity of open field test to determine locomotor activity. The overall blue trace shows the LRS. The genome‐wide QTL map showing suggestive QTLs on Chromosome 6, 9, 10, 15 and 18 on open field parameters. No QTL identified in velocity parameter. The lower gray horizontal line represents suggestive LRS genome‐wide thre [file GBB-24-e70014-s002.pdf]
