# Supplementary material for: Investigating Motor Coordination Using BXD Recombinant Inbred Mice to Model the Genetic Underpinnings of Developmental Coordination Disorder
Source: Genes Brain Behav. 2025 Mar 12;24(2):e70014. doi: 10.1111/gbb.70014 (PMC11898013; doi:10.1111/gbb.70014)
Supplement: Supplementary file 2 — Tables S1‐S3. Supporting Information. [file GBB-24-e70014-s001.docx]

**Table S1.** Summary of all variants

| **Chromosome** | **Position** | **File** | **N variants** | **Splice donor variant** | **Splice acceptor variant** | **Stop gained** | **Frameshift variant** | **Stop lost** | **Start lost** | **Inframe insertion** | **Inframe deletion** | **Protein altering variant** | **Missense variant** | **Splice region variant** | **Synonymous variant** | **Coding sequence variant** | **Mature miRNA variant** | **5 prime UTR variant** | **3 prime UTR variant** | **Non coding transcript exon variant** | **Intron variant** | **Upstream gene variant** | **Downstream gene variant** | **Intergenic variant** |
| --- | --- | --- | --- | --- | --- | --- | --- | --- | --- | --- | --- | --- | --- | --- | --- | --- | --- | --- | --- | --- | --- | --- | --- | --- |
| Chr 10 | 100.9- 104 | File 1 | 8,714.00 | 0 | 0 | 0 | 0 | 0 | 0 | 0 | 0 | 0 | 0 | 4 | 4 | 0 | 0 | 1 | 19 | 80 | 1966 | 394 | 545 | 5,699 |
| Chr 12 | 75 - 79 | File 2 | 2,930.00 | 1 | 0 | 0 | 0 | 0 | 0 | 1 | 0 | 0 | 30 | 9 | 47 | 0 | 0 | 6 | 32 | 3 | 2408 | 57 | 136 | 200 |
| Chr 17 | 40-48 | File 3 | 21,049.00 | 1 | 0 | 2 | 1 | 0 | 0 | 0 | 0 | 0 | 36 | 11 | 66 | 0 | 0 | 33 | 167 | 128 | 7766 | 985 | 618 | 11235 |
| Chr 19 | 30 - 33 | File 4 | 3,066.00 | 0 | 0 | 0 | 0 | 0 | 0 | 0 | 0 | 0 | 3 | 1 | 8 | 0 | 0 | 12 | 61 | 23 | 1426 | 192 | 238 | 1102 |
| Chr 2 | 116.5-118.9 | File 5 | 8,656.00 | 1 | 0 | 1 | 0 | 0 | 0 | 0 | 0 | 0 | 44 | 14 | 81 | 0 | 0 | 30 | 205 | 164 | 5381 | 706 | 227 | 1802 |
| Chr 3 | 36.25-37.87 | File 6 | 12,831.00 | 4 | 1 | 0 | 0 | 0 | 0 | 2 | 2 | 0 | 32 | 20 | 84 | 0 | 0 | 68 | 151 | 540 | 5256 | 1182 | 788 | 4701 |
| Chr 3 | 36.55 - 37.9 | File 7 | 12,903.00 | 4 | 1 | 0 | 0 | 0 | 0 | 2 | 2 | 0 | 32 | 20 | 84 | 0 | 0 | 68 | 151 | 542 | 5262 | 1060 | 767 | 4908 |
| Chr 4 | 25 - 32.1 | File 8 | 4,813.00 | 0 | 0 | 0 | 0 | 0 | 0 | 0 | 0 | 0 | 0 | 2 | 8 | 0 | 0 | 0 | 20 | 23 | 531 | 117 | 206 | 3,906 |
| Chr 5 | 51.5 - 52.95 | File 9 | 2,537.00 | 0 | 0 | 0 | 0 | 0 | 0 | 0 | 0 | 0 | 3 | 3 | 5 | 0 | 0 | 2 | 12 | 83 | 556 | 253 | 195 | 1425 |
| Chr 6 | 63.8 - 64.8 | File 10 | 2,004.00 | 0 | 0 | 0 | 0 | 0 | 0 | 0 | 0 | 0 | 1 | 0 | 3 | 0 | 0 | 0 | 13 | 15 | 1812 | 10 | 92 | 58 |
| Chr 6 | 98 - 114 | File 11 | 47,853.00 | 0 | 0 | 0 | 1 | 0 | 0 | 0 | 0 | 0 | 20 | 26 | 90 | 0 | 0 | 23 | 192 | 435 | 18454 | 1831 | 1647 | 25134 |
| Chr 8 | 24.8 - 42.7 | File 12 | 137,548.00 | 7 | 1 | 1 | 10 | 1 | 1 | 2 | 2 | 2 | 232 | 86 | 310 | 0 | 2 | 191 | 968 | 2386 | 61313 | 6669 | 6641 | 58721 |
| Chr 9 | 77.9-99.8 | File 13 | 60,314.00 | 1 | 3 | 0 | 0 | 0 | 0 | 2 | 1 | 0 | 74 | 55 | 130 | 1 | 0 | 100 | 363 | 1148 | 24251 | 3315 | 3101 | 27769 |
| Chr 9 | 80.5-94.5 | File 14 | 44,467.00 | 0 | 2 | 0 | 0 | 0 | 0 | 1 | 0 | 0 | 43 | 37 | 77 | 1 | 0 | 35 | 169 | 862 | 15012 | 2393 | 2366 | 23469 |
| Chr 9 | 81-95 | File 15 | 44,583.00 | 0 | 2 | 0 | 0 | 0 | 0 | 1 | 0 | 0 | 43 | 37 | 80 | 1 | 0 | 35 | 185 | 875 | 15359 | 2385 | 2378 | 23202 |

**Table S2.** Summary of QTL analysis by sex

|  |  | **COMBINED SEXES** | | | | | | | | **MALE ONLY** | | | | | | | | **FEMALE ONLY** | | | | | | | |
| --- | --- | --- | --- | --- | --- | --- | --- | --- | --- | --- | --- | --- | --- | --- | --- | --- | --- | --- | --- | --- | --- | --- | --- | --- | --- |
| **GeneNetwork ID** | **Phenotype** | **Peak chromosome** | **Peak position (Mb)** | **QTL interval (Mb)** | **Peak marker** | **Linkage (LRS)** | **Significance threshold** | **Suggestive threshold** | **Significant or suggestive?** | **Peak chromosome** | **Peak position (Mb)** | **QTL interval (Mb)** | **Peak marker** | **Linkage (LRS)** | **Significance threshold** | **Suggestive threshold** | **Significant or suggestive?** | **Peak chromosome** | **Peak position (Mb)** | **QTL interval (Mb)** | **Peak marker** | **Linkage (LRS)** | **Significance threshold** | **Suggestive threshold** | **Significant or suggestive?** |
| BXD_20969 | Body speed | Chr 8 | 41.378 | 18.064-75.589 | rs3167597 | 13.878 | 13.88 | 11.36 | SUGGESTIVE | Chr 9 | 98.544-106.018 | 94.448-114.714 | rs33725243 | 11.358 | 13.62 | 10.59 | SUGGESTIVE | Chr 8 | 41.378 | 18.064-43.371 | rs3167597 | 14.43 | 14.36 | 10.73 | SIGNIFICANT |
| BXD_20969 | Body speed | Chr 19 | 11.865 | 26.545-54.298 | - | 11.865 | 13.88 | 11.36 | SUGGESTIVE |  |  |  |  |  |  |  |  |  |  |  |  |  |  |  |  |
| BXD_21408 | Leg combination | Chr 4 | 25.58-32.02 | 24.736-32.082 | rsm10000001920 | 20.768 | 20.77 | 15.19 | SUGGESTIVE | Chr 4 | 25.58-32.02 | 24.736-32.082 | rsm10000001920 | 17.249 | 17.25 | 13.54 | SUGGESTIVE | Chr 4 | 25.58-32.02 | 24.736-32.082 | rsm10000001920 | 21.404 | 22.44 | 14.36 | SUGGESTIVE |
| BXD_21408 | Leg combination | Chr 16 | 89.834-97.524 | 89.83-97.578 | rs258510349 | 20.768 | 20.77 | 15.19 | SUGGESTIVE | Chr 16 | 89.834-97.524 | 89.83-97.578 | rs258510349 | 17.249 | 17.25 | 13.54 | SUGGESTIVE | Chr 16 | 89.834-97.524 | 89.83-97.578 | rs258510349 | 21.404 | 22.44 | 14.36 | SUGGESTIVE |
| BXD_21408 | Leg combination | Chr 19 | 29.734 | 29.734-32.43 |  | 15.831 | 20.77 | 15.19 | SUGGESTIVE | Chr 19 | 29.734 | 26.545-32.43 |  | 14.722 | 17.25 | 13.54 | SUGGESTIVE | Chr19 | 29.734 | 29.734-32.43 |  | 16.019 | 22.44 | 14.36 | SUGGESTIVE |
| BXD_20967 | Duty factor | Chr 19 | 29.734 | 26.545-32.43 |  | 16.576 | 15.53 | 14.05 | SIGNIFICANT | Chr 4 | 25.58-32.02 | 21.683-40.278 | rsm10000001920 | 11.454 | 12.95 | 11.45 | SUGGESTIVE | Chr 19 | 29.734 | 26.545-32.43 |  | 14.252 | 14.24 | 12.77 | SIGNIFICANT |
| BXD_20967 | Duty factor |  |  |  |  |  |  |  |  | Chr16 | 89.834-97.524 | 87.844-97.578 | rs258510349 | 11.454 | 12.95 | 11.45 | SUGGESTIVE | Chr 10 | 3.103-21.021 | 0-24.382 | rsm10000006866 | 12.797 | 14.24 | 12.77 | SUGGESTIVE |
| BXD_21427 | Step cycle | Chr 3 | 36.533-37.62 | 35.722-37.863 | rsm10000001570 | 18.497 | 18.5 | 14.9 | SUGGESTIVE | Chr 4 | 124.386-124.84 | 124.208-125.588 | rs27553983 | 15.737 | 17.75 | 11.38 | SUGGESTIVE | Chr 4 | 124.386-124.84 | 124.208-125.588 | rs27553983 | 14.294 | 14.45 | 13.21 | SUGGESTIVE |
| BXD_21427 | Step cycle | Chr 5 | 66.613-73.762 | 66.584-73.826 | rsm10000002587 | 14.9 | 18.5 | 14.9 | SUGGESTIVE |  |  |  |  |  |  |  |  |  |  |  |  |  |  |  |  |
| BXD_20971 | Stance duration | Chr 3 | 36.533-37.62 | 35.722-37.863 | rsm10000001570 | 15.809 | 16.73 | 14.71 | SUGGESTIVE | Chr 4 | 124.386-124.84 | 124.208-125.588 | rs27553983 | 16.198 | 16.43 | 11.96 | SUGGESTIVE | Chr 4 | 124.386-124.84 | 124.208-125.588 | rs27553983 | 14.638 | 14.71 | 13.34 | SUGGESTIVE |
| BXD_20972 | Swing duration | Chr 15 | 95.163-95.209 | 95.148-95.234 | rsm10000012232 | 21.014 | 21.73 | 13.5 | SUGGESTIVE | Chr 15 | 95.163-95.209 | 88.259-95.234 | rsm10000012232 | 15.052 | 15.05 | 11.86 | SIGNIFICANT | Chr 15 | 95.163-95.209 | 95.148-95.234 | rsm10000012232 | 26.683 | 26.68 | 14.38 | SIGNIFICANT |
| BXD_20972 | Swing duration |  |  |  |  |  |  |  |  | Chr 6 | 143.002-149.266 | 137.714-149.266 | rsm10000004002 | 12.028 | 15.05 | 11.86 | SUGGESTIVE |  |  |  |  |  |  |  |  |
| BXD_20972 | Swing duration |  |  |  |  |  |  |  |  | Chr X | 93.826 | 73.429-95.233 | rsm10000014501 | 12.028 | 15.05 | 11.86 | SUGGESTIVE |  |  |  |  |  |  |  |  |
| BXD_21410 | Posterior extreme position | Chr 6 | 98.288-103.719 | 97.984-114.013 | rsm10000003421 | 15.581 | 15.58 | 11.36 | SIGNIFICANT | Chr 4 | 124.386-124.84 | 124.208-125.588 | rs27553983 | 19.799 | 19.91 | 9.33 | SUGGESTIVE | NA |  |  |  |  |  |  | Non-significant |
| BXD_20086 | Baseline performance | NA |  |  |  |  | 15.91 | 12.26 | Non-significant | Chr 5 | 51.546 | 45.179-52.439 | rs3704725 | 22.109 | 20.53 | 14.36 | SIGNIFICANT | NA |  |  |  |  | 11.36 | 7.98 | Non-significant |
| BXD_20086 | Baseline performance |  |  |  |  |  |  |  |  | Chr 7 | 40.415-44.318 | 40.415-44.325 | rsm10000004419 | 15.899 | 20.53 | 14.36 | SUGGESTIVE |  |  |  |  |  |  |  |  |
| BXD_20094 | Performance Improvement | Chr 4 | 33.861 | 32.02-40.278 |  | 14.537 | 17.41 | 14.2 | SUGGESTIVE | Chr 13 | 29-33.749 | 28.687-38.153 | rsm10000009646 | 17.12 | 17.12 | 14.74 | SIGNIFICANT | Chr 12 | 48.803-54.193 | 47.767-54.574 | rsm10000009271 | 41.532 | 41.53 | 11.24 | SIGNIFICANT |
| BXD_20094 | Performance Improvement | Chr 12 | 75.182-79.237 | 75.122-104.629 |  | 14.465 | 17.41 | 14.2 | SUGGESTIVE | Chr 5 | 44.121-45.179 | 41.566-51.546 | rsm10000002332 | 14.743 | 17.12 | 14.74 | SUGGESTIVE | Chr 12 | 59.376-64.388 | 45.289-84.105 | rs3664781 | 14.965 | 41.53 | 11.24 | SUGGESTIVE |
| BXD_20094 | Performance Improvement |  |  |  |  |  |  |  |  |  |  |  |  |  |  |  |  | Chr 15 | 92.9-94.237 | 83.044-95.807 | rsm10000012194 | 12.605 | 41.53 | 11.24 | SUGGESTIVE |
| BXD_20362 | Distance travelled | Chr 6 | 61.638-64.713 | 52.062-64.802 | rs30223784 | 10.174 | 11.73 | 10.17 | SUGGESTIVE | NA |  |  |  |  | 17.2 | 12.99 | Non-significant | Chr 11 | 107.053-107.925 | 104.604-110.157 | rsm10000008806 | 12.494 | 15.91 | 11.4 | SUGGESTIVE |
| BXD_20365 | Center Time | Chr 9 | 81.294-94.662 | 81.294-94.742 | rsm10000006852 | 17.649 | 17.68 | 16.3 | SUGGESTIVE | Chr 9 | 81.294-94.662 | 78.047-98.544 | rsm10000006852 | 10.883 | 12.95 | 10.8 | SUGGESTIVE | Chr 10 | 107.483-108.525 | 107.476-110.399 | rs29327089 | 17.566 | 18.21 | 16.61 | SUGGESTIVE |
| BXD_20365 | Center Time | Chr 10 | 109.298-109.379 | 107.476-110.399 | rsm10000007316 | 17.649 | 17.68 | 16.3 | SUGGESTIVE |  |  |  |  |  |  |  |  | Chr 9 | 94.45 | 78.047-98.544 | rsm10000006502 | 16.764 | 18.21 | 16.61 | SUGGESTIVE |
| BXD_20363 | Peripheral Time | Chr 9 | 81.294-94.662 | 78.047-98.544 | rsm10000006852 | 12.93 | 18.82 | 12.93 | SUGGESTIVE |  |  |  |  |  | 16.23 | 11.15 | Non-significant |  |  |  |  |  | 17.35 | 13.14 | Non-significant |
| BXD_20363 | Peripheral Time | Chr 10 | 109.298-109.379 | 107.476-110.399 | rsm10000007316 | 12.93 | 18.82 | 12.93 | SUGGESTIVE |  |  |  |  |  |  |  |  |  |  |  |  |  |  |  |  |
| BXD_20367 | Time moving | Chr 9 | 94.609-94.609 | 78.047-106.018 | rsm10000006505 | 17.449 | 16.74 | 13.22 | SIGNIFICANT | Chr 9 | 94.609-94.609 | 78.047-98.544 | rsm10000006505 | 15.117 | 14.81 | 12.85 | SIGNIFICANT | Chr 9 | 98.544-106.018 | 78.047-106.018 | rs33725243 | 16.556 | 16.56 | 14.41 | SUGGESTIVE |
| BXD_20367 | Time moving | Chr 18 | 3.179-8.492 | 0-9.341 | rsm10000013285 | 16.742 | 16.74 | 13.22 | SIGNIFICANT | Chr 10 | 110.399-116.134 | 108.525-120.593 | rsm10000007317 | 14.523 | 14.81 | 12.85 | SUGGESTIVE | Chr 18 | 3.179-8.492 | 0-9.641 | rsm10000013285 | 15.622 | 16.56 | 14.41 | SUGGESTIVE |
| BXD_20367 | Time moving |  |  |  |  |  |  |  |  | Chr 18 | 3.179-8.492 | 0-9.641 | rsm10000013285 | 13.517 | 14.81 | 12.85 | SUGGESTIVE | Chr 15 | 13.707-18.877 | 13.556-22.164 | rsm10000011697 | 14.598 | 16.56 | 14.41 | SUGGESTIVE |
| BXD_20369 | Time Not Moving | Chr 9 | 94.609-94.609 | 78.047-106.018 | rsm10000006505 | 14.63 | 14.79 | 13.1 | SUGGESTIVE | Chr 10 | 116.64-120.587 | 107.476-120.593 | rs29316701 | 11.956 | 13.21 | 11.93 |  | Chr 9 | 98.544-106.018 | 78.047-106.018 | rs33725243 | 16.67 | 16.68 | 13.05 | SUGGESTIVE |
| BXD_20369 | Time Not Moving | Chr 18 | 3.179-8.492 | 0-9.641 | rsm10000013285 | 14.571 | 14.79 | 13.1 | SUGGESTIVE | Chr 15 | 14.303-17.393 | 13.556-22.164 | rsm10000011708 | 11.956 | 13.21 | 11.93 |  | Chr 18 | 3.179-8.492 | 0-9.641 | rsm10000013285 | 15.709 | 16.68 | 13.05 | SUGGESTIVE |
| BXD_20369 | Time Not Moving | Chr 15 | 13.707-18.877 | 13.556-22.164 | rsm10000011697 | 13.102 | 14.79 | 13.1 | SUGGESTIVE |  |  |  |  |  |  |  |  | Chr 15 | 13.707-14.297 | 13.556-18.877 | rsm10000011697 | 14.612 | 16.68 | 13.05 | SUGGESTIVE |
| BXD_20371 | Velocity |  |  |  |  |  | 18.59 | 13.76 |  | Chr 6 | 114.013-114.055 | 114.01-114.056 | rsm10000004190 | 13.74 | 14.5 | 13.3 |  | Chr 4 | 96.669-100.81 | 96.265-101.394 | rsm10000002024 | 23.855 | 23.86 | 10.09 | SUGGESTIVE |
| BXD_20371 | Velocity |  |  |  |  |  |  |  |  | Chr 5 | 113.383-116.339 | 104.083-116.407 | rs36795578 | 13.438 | 14.5 | 13.3 |  | Chr 5 | 104.085-113.316 | 104.083-113.383 | rsm10000002769 | 23.855 | 23.86 | 10.09 | SUGGESTIVE |
| BXD_20358 | Cliff aversion | Chr 2 | 116.64-118.87 | 99.794-122.467 | rsm10000001485 | 13.011 | 14.79 | 11.82 | SUGGESTIVE |  |  |  |  |  | 12.73 | 10.17 | Non-significant | Chr 4 | 96.265 | 96.265-101.394 |  | 16.031 | 15.01 | 13.51 | SIGNIFICANT |
| BXD_20358 | Cliff aversion | Chr 10 | 110.399-116.134 | 108.525-116.64 | rsm10000007317 | 12.037 | 14.79 | 11.82 | SUGGESTIVE |  |  |  |  |  |  |  |  | Chr 5 | 104.083 | 104.083-113.383 | | 14.812 | 15.01 | 13.51 | SUGGESTIVE |
| BXD_20360 | Forelimb grasp | Chr 10 | 102.375-103.37 | 97.65-103.513 | rs13480734 | 18.227 | 19.96 | 14.81 | SUGGESTIVE | Chr 13 | 48.253 | 28.687-54.669 |  | 11.66 | 12.81 | 11.35 | SUGGESTIVE | Chr 12 | 54.574-57.951 | 54.193-57.951 | rsm10000009280 | 12.884 | 18.81 | 12.71 | SUGGESTIVE |
| BXD_20357 | Negative geotaxis |  |  |  |  |  | 13.35 | 12.15 | Non-significant | Chr 6 | 12.76-14.361 | 0-52.293 | rsm10000003148 | 11.786 | 13.52 | 11.02 | SUGGESTIVE | Chr 2 | 118.871-121.551 | 118.87-121.551 | rsm10000001330 | 14.473 | 20.34 | 13.45 | SUGGESTIVE |
| BXD_20359 | Surface righting | Chr 17 | 40.571-48.495 | 39.789-48.6 | rs13482981 | 18.281 | 19.47 | 14.91 | SUGGESTIVE | Chr 13 | 26.079-28.687 | 23.934-29 | rsm10000009727 | 20.218 | 20.22 | 15.27 | SUGGESTIVE | Chr 17 | 40.571-48.495 | 39.789-48.6 | rs13482981 | 25.359 | 25.36 | 16.34 | SUGGESTIVE |
| BXD_20359 | Surface righting |  |  |  |  |  |  |  |  | Chr 8 | 95.693-102.72 | 95.678-111.319 | rsm10000005699 | 18.228 | 20.22 | 15.27 | SUGGESTIVE |  |  |  |  |  |  |  |  |

Haley-Knot mapping results for phenotypes with either the combined mean (columns 3-10), only males (columns 11-18) or only females (columns 19-26). Columns 1 gives the GeneNetwork ID for the trait, and column 2 gives a shortform description. Peak chromosome and peak position give the location of the most significant association, and linkage gives the likelihood ratio statistic (LRS) for the association. The thresholds were calculated by 5000 permutations of the data, with significant being p < 0.05, and suggestive being p < 0.63 (one false positive per genome scan).

**Table S3. Identification of priority genes using three criteria.**

| **Gene** | **Criterion A**  **Expression**  **(n = 59 genes)** | **Criterion B**  **Function**  **(n = 7)** | **Criterion C Polymorphism**  **(n = 3)** |
| --- | --- | --- | --- |
| Fut9 | Brain | _ | _ |
| Map3k7 | Brain and/or Muscle | _ | _ |
| Fhl5 | Brain and/or Muscle | _ | _ |
| Gpr63 | Brain and/or Muscle | Yes | _ |
| Ufl1 | Brain and/or Muscle | _ | _ |
| Spry1 | Brain and/or Muscle | _ | _ |
| Spata5 | Brain and/or Muscle | Yes | Yes |
| Nudt6 | Brain and/or Muscle | _ | _ |
| Fgf2 | Brain and/or Muscle | _ | _ |
| Bbs12 | Brain and/or Muscle | _ | _ |
| Cetn4 | Brain and/or Muscle | _ | _ |
| Il21 | None | _ | _ |
| Il2 | None | _ | _ |
| Adad1 | None | _ | _ |
| Trpc3 | Brain and/or Muscle | Yes | _ |
| Bbs7 | Brain and/or Muscle | _ | _ |
| Anxa5 | Brain and/or Muscle | _ | _ |
| Cntn4 | Brain and/or Muscle | _ | _ |
| Mitf | Brain and/or Muscle | _ | _ |
| Cntn3 | Brain and/or Muscle | _ | _ |
| Foxp1 | Brain and/or Muscle | _ | _ |
| Pdzrn3 | Brain and/or Muscle | _ | _ |
| Ppp4r2 | Brain and/or Muscle | _ | _ |
| Cntn6 | Brain | yes | Yes |
| Rybp | Brain and/or Muscle | _ | _ |
| Shq1 | Brain and/or Muscle | _ | _ |
| Chl1 | Brain and/or Muscle | yes | Yes |
| Glt8d4 | Brain and/or Muscle | _ | _ |
| Gxylt2 | Brain and/or Muscle | _ | _ |
| Eif4e3 | Brain and/or Muscle | _ | _ |
| Grm7 | Brain | yes | _ |
| Srgap3 | Brain and/or Muscle | _ | _ |
| Lmcd1 | Brain and/or Muscle | _ | _ |
| Setd5 | Brain and/or Muscle | _ | _ |
| Cpne9 | Brain and/or Muscle | _ | _ |
| Rad18 | Brain and/or Muscle | _ | _ |
| Lhfpl4 | Brain | _ | _ |
| Brpf1 | Brain and/or Muscle | _ | _ |
| Mtmr14 | Brain and/or Muscle | _ | _ |
| Cidec | Brain and/or Muscle | _ | _ |
| Arpc4 | Brain and/or Muscle | _ | _ |
| Tada3l | Brain and/or Muscle | _ | _ |
| Tada3 | Brain and/or Muscle | _ | _ |
| Camk1 | Brain and/or Muscle | _ | _ |
| Ttll3 | Brain and/or Muscle | _ | _ |
| Ogg1 | Brain and/or Muscle | yes | _ |
| Il17rc | Brain and/or Muscle | _ | _ |
| Rpusd3 | Brain and/or Muscle | _ | _ |
| Fancd2 | Brain and/or Muscle | _ | _ |
| Thumpd3 | Brain and/or Muscle | _ | _ |
| Irak2 | Brain and/or Muscle | _ | _ |
| Jagn1 | Brain and/or Muscle | _ | _ |
| Ssu2 | Brain and/or Muscle | _ | _ |
| Il17re | Brain and/or Muscle | _ | _ |
| Prrt3 | Brain and/or Muscle | _ | _ |
| Cav3 | Brain and/or Muscle | _ | _ |
| Tmem111 | Brain and/or Muscle | _ | _ |
| Emc3 | Brain and/or Muscle | _ | _ |
| Brk1 | Brain and/or Muscle | _ | _ |

Empty areas show genes that did not meet the criterion system.
